# Genome-wide maps of UV damage repair and mutation suppression by CPD photolyase

**DOI:** 10.1093/nar/gkaf495

**Published:** 2025-06-11

**Authors:** Kaitlynne A Bohm, Marian F Laughery, Piotr A Mieczkowski, Steven A Roberts, John J Wyrick

**Affiliations:** School of Molecular Biosciences, Washington State University, Pullman, WA 99164, United States; School of Molecular Biosciences, Washington State University, Pullman, WA 99164, United States; Department of Genetics, Lineberger Comprehensive Cancer Center, University of North Carolina, Chapel Hill, NC 27599, United States; School of Molecular Biosciences, Washington State University, Pullman, WA 99164, United States; Department of Microbiology and Molecular Genetics, University of Vermont Cancer Center, University of Vermont, Burlington, VT 05405, United States; School of Molecular Biosciences, Washington State University, Pullman, WA 99164, United States

## Abstract

Ultraviolet (UV) light causes cyclobutane pyrimidine dimers (CPDs) and other DNA lesions that must be efficiently repaired to prevent cell death and mutagenesis. While mammals utilize the nucleotide excision repair (NER) pathway to repair CPDs, many species primarily utilize photolyase enzymes to repair UV damage. Our understanding of how different genomic and chromatin features impact photolyase repair across a eukaryotic genome is limited. Here, we map repair of CPDs by photolyase across the yeast genome at single-nucleotide resolution. Our data indicate that yeast photolyase repairs CPDs more rapidly than NER, but photolyase activity is inhibited at certain classes of transcription factor binding sites and in nucleosomes. Repair in nucleosomes is particularly inhibited when CPDs are located along the 3′ side of the nucleosomal DNA or at minor-in rotational settings. Our data indicate that photolyase efficiently repairs the non-transcribed strand of yeast genes, but repair of the transcribed strand (TS) is inhibited. Genome-wide analysis of UV-induced mutations in NER-deficient photoreactivated yeast reveals a striking enrichment of mutations along the TS of yeast genes. These data indicate that inhibition of photolyase repair along the TS, likely due to occlusion of CPDs by RNA polymerase II stalling, promotes UV mutagenesis.

## Graphical Abstract

**Graphical Abstract:**
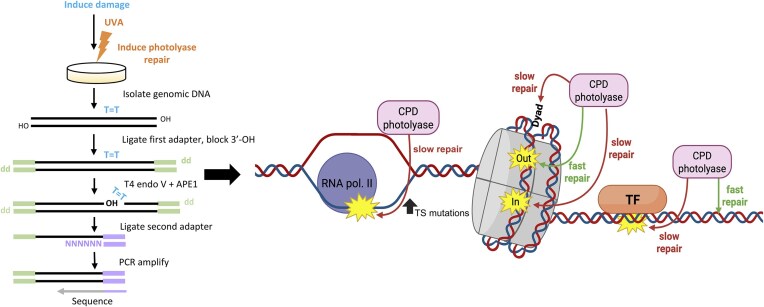

## Introduction

UV light is a ubiquitous hazard for all terrestrial species because it induces bulky, helix-distorting lesions within cellular DNA. The primary class of UV-induced DNA lesions are cyclobutane pyrimidine dimers (CPDs), which form between neighboring pyrimidine bases and constitute ∼80–90% of UV damage [1]. In order to avoid the mutagenic and cytotoxic effects of CPDs and other UV-induced DNA damage (e.g. 6–4 pyrimidine-pyrimidone photoproducts [6–4PPs]), these lesions must be efficiently repaired. For placental mammals, the primary mechanism for repairing CPDs and other UV-induced lesions is the nucleotide excision repair (NER) pathway [2]. NER is initiated by either direct detection of helix-distorting DNA lesions throughout the genome, a pathway known as global genomic-NER (GG-NER) [2], or upon RNA polymerase II stalling at a lesion, which triggers a pathway known as transcription coupled-NER (TC-NER) [3]. After damage detection, NER enzymes must work in concert to unwind the DNA, verify the lesion, excise a DNA fragment containing the lesion and as much as 24–30 nt of flanking DNA, resynthesize this DNA, and ligate the newly replicated DNA to complete repair [2]. These repair steps are not just energetically costly, but the process of NER can also be intrinsically mutagenic, particularly when repairing closely spaced DNA lesions on opposing DNA strands [4, 5].

Many species can also repair CPD lesions through a distinct pathway mediated by a single repair enzyme known as CPD photolyase [6, 7]. CPD photolyases are flavin adenine dinucleotide (FAD)-dependent enzymes that use light to directly reverse CPD damage in an efficient and error-free mechanism known as photoreactivation [6–8]. Photoreactivation by CPD photolyases was the first DNA repair pathway identified [9], and the CPD photolyase reaction mechanism has been extensively studied (e.g. [6, 10]). Photolyases recognize damaged DNA by interacting with the negatively charged DNA backbone flanking the lesion and flipping the damaged bases into an active site cavity [6, 7, 11]. Upon activation by long wavelength UV (e.g. UVA: 320–400 nm) or blue light, the photolyase transfers a radical electron from excited FAD to cleave the cyclobutane ring of the CPD and restore the damaged bases to their monomeric, undamaged form [6, 7]. CPD photolyases are found not only in bacteria and archaea, but also fungi, plants, invertebrates, and many vertebrates, including marsupials but not placental mammals, essentially all of which also have a functional NER pathway [6, 7, 12, 13]. Despite intensive study [14–19], how CPD photolyase and NER cooperate to repair damage and prevent UV-induced mutations in the myriad of different genomic and chromatin contexts that comprise a eukaryotic genome still remains unclear.

Our understanding of how the NER pathway repairs CPD lesions in different genomic and chromatin contexts has been revolutionized by genome-wide maps of repair in UV-irradiated yeast and human cells [20–25]. For example, these studies have revealed that DNA-bound transcription factors (TFs) inhibit GG-NER in both yeast and human cells [22, 26–28], resulting in elevated mutation density at TF-binding sites (TFBS) in human melanomas [26, 28]. Packaging of DNA with histone proteins into nucleosomes has also been found to inhibit GG-NER throughout the genome. CPDs are repaired by NER more slowly near the central nucleosome dyad in both yeast and human cells [22, 29], and this repair inhibition is asymmetric, with the 3′ side of the nucleosomal DNA having slower repair and elevated mutation density in skin cancers [21]. Finally, genome-wide repair maps have revealed faster repair of the transcribed strand (TS) of eukaryotic genes by the TC-NER pathway, and revealed the roles of canonical and novel TC-NER factors responsible for efficient repair of transcribed DNA [22, 24, 25,30–33]. Previous studies of CPD photolyase repair in yeast at individual genes or genomic regions have suggested that photolyase repair activity is also impacted by DNA bound proteins and transcription (e.g. [15–19, 34, 35–37]). However, despite the critical importance of CPD photolyase for UV resistance in most species, photolyase repair of CPD lesions has yet to be analyzed using genome-wide methods.

Here, we used our CPD-seq method [22, 38] to map repair of CPD lesions by yeast photolyase in both NER-proficient and -deficient cells. Our data reveal that CPD photolyase more efficiently repairs CPDs than NER, but its repair activity is inhibited in nucleosomes and at DNA-binding sites of specific classes of TFs. Photolyase repair activity is also inhibited along the TS of nearly all yeast genes. Genomic sequencing of NER-deficient yeast that were repeatedly exposed to UV light and photoreactivated following UV irradiation revealed a striking enrichment of UV-induced mutations along the TS of yeast genes, consistent with the observed inhibition of photolyase repair activity. Finally, our data indicate that in NER-proficient cells, the NER pathway promotes repair in genomic and chromatin contexts (i.e. TFBS, nucleosomes, and TS of genes) that are normally refractory to repair by photolyase.

## Materials and methods

### Yeast strains

The *rad14*Δ mutant was previously constructed by PCR mediated-deletion [39] using the *TRP1* marker to delete *RAD14* in MP019 (BY4741 background, MATa *ura3Δ leu2Δ met15Δ his3Δ1 trp1::HIS3*) to generate MP074. To generate the *rad14*Δ *phr1*Δ double mutant (KB017), the *rad14*Δ mutant was transformed with a *LEU2* knockout construct to delete *PHR1* (yeast CPD photolyase). An NER-deficient haploid yeast strain expressing the *Drosophila melanogaster* [6, 4] photolyase (dPhr6-4) was constructed by integration of the plasmid pML157 into MP074 (i.e. *rad14*Δ) to generate YML467. pML157 was constructed by isolation of the *Drosophila* photolyase gene from p64PLA5 (Addgene #67 284) via NheI/XhoI digestion and gel purification, and ligation with the p405TEF1 (Addgene #15 968), which had previously been SpeI/XhoI digested and gel purified. The *rad14Δ* yeast strain was then transformed with pML157 and integration was selected for using synthetic complete media plates lacking leucine (SC-LEU). The presence of the dPhr6-4 gene was confirmed by PCR screening, and the plasmid was found to have integrated at the yeast *TEF1* promoter. For passaging assays, a diploid *rad14Δ* strain expressing dPhr6-4 (i.e. YML480) was constructed by mating YML467 with YML477, a *rad14Δ* mutant of BY4742, and screening on selective media.

### UV sensitivity assay

Haploid yeast strains were grown in Yeast extract Peptone Dextrose (YPD) media overnight, serially diluted tenfold, spotted onto rich media plates, and allowed to dry. Plates were exposed to approximately 6 or 12.5 J/m^2^ of UVC light (based on our previous calibration), followed by 0 min photoreactivating UVA light (-PR) or 40 min photoreactivating UVA light (+PR) using a Spectroline EA-160 lamp. Plates were then incubated at 30°C in the dark until growth appeared about 2–3 days later.

### Alkaline gel assay of bulk repair of CPD lesions

WT (BY4741), *rad14Δ*, or *rad14Δ phr1Δ* mutant yeast cells were grown in 50mL of YPD medium until they reached mid-log phase (OD600 ∼0.65). Cells were pelleted by centrifugation and resuspended in sterile dH2O. Of this, 10mL of cells were collected for a “No UV” control, and the remaining cells were exposed to 100J/m2 of UVC light (based on our previous calibration). Cells were collected immediately following UV exposure to serve as a “0 h UV” timepoint, and the remaining cells were pelleted by centrifugation, resuspended in 30 mL of 2% glucose solution, poured in a petri dish, and allowed to repair for 0.5, 1, or 2 h under UVA (peak ∼365 nm) photoreactivating light. Cells were pelleted by centrifugation and cell pellets were stored at −80°C until DNA isolation. Genomic DNA was isolated from yeast by vortexing with acid washed glass beads in lysis buffer (2% Triton-X 100, 1% SDS, 100 mM NaCl, 10 mM Tris-HCl pH 8, 1 mM EDTA), followed by phenol:chloroform:isoamylalcohol (PCI, 25:24:1) extraction and precipitation with ethanol, and then RNase A treated for ≥15 min at 37°C. Genomic DNA concentration was measured using a NanoDrop spectrophotometer and aliquots of each sample (5 μg of DNA) were treated with T4 endonuclease V or mock treated for 2 h at 37°C to cleave DNA at sites of CPD lesions. The T4 endoV digestion was stopped by addition of 6x alkaline loading dye and run on a 1.2% alkaline gel at 30V for 20 h to denature and separate the nicked single-stranded DNA. The gel was neutralized for 1 h in neutralization buffer (1M Tris-HCl, 1.5M NaCl, pH 7.5), stained with SYBR gold (Thermo Fisher Scientific) for 1 h, and washed twice with dH2O, 1 h per wash. The stained gel was then analyzed via Typhoon FLA biomolecular imager (GE Healthcare). ImageQuant v5.2 was used to determine the intensity of each lane. The median intensity of each lane was used to determine the average fragment size based on known fragment sizes of the HindIII-digested λ phage DNA ladder and the distance of band migration. A Poisson distribution was used to determine the approximate number of CPDs per kb at each timepoint, and each timepoint was compared to the “0 h UV” timepoint, as previously described [40, 41]. The resulting repair data were analyzed using GraphPad Prism.

### Statistical analysis

Significance (*P*-values) for alkaline gel analysis was calculated using an unpaired t-test with Holm–Sidak correction for multiple hypothesis testing. Each experimental sample (e.g. WT + PR, etc.) had three biological replicates, with the exception of *rad14*Δ*phr1*Δ +PR, which had four replicates. Statistical significance (*P*-values) of the difference in the fraction of unrepaired CPDs along the TS and NTS of yeast genes was determined by comparing the aggregate fraction of unrepaired CPDs for the six bins in the transcribed regions of yeast genes using a paired t-test.

### CPD-seq of photoreactivated yeast

We modified our published CPD-seq [22, 38] to include photoreactivation of yeast cells by the endogenous CPD photolyase. Briefly, WT or *rad14Δ* yeast cultures were grown to an OD_600_ of approximately 0.8 in YPD, pelleted, and resuspended in dH_2_O. Unexposed cells were collected for a “No UV” sample, and the remaining cells were treated with 125 J/m^2^ of UVC light based on our previous calibration. Approximately one third of the cells were collected immediately following UV exposure (“0 min UV”), and the remaining cells were resuspended in 2% glucose, placed in a petri dish, and allowed to repair under UVA photoreactivating light for 30 or 60 min at room temperature. Genomic DNA was then isolated from yeast via vortexing with acid-washed glass beads, followed by PCI extraction and ethanol precipitation, and the resulting genomic DNA was treated with RNaseA for at least 30 min. DNA was sonicated into fragments between ∼200 and 700 bp in length using a Bioruptor 300 Sonicator (15 cycles: 30 s on, 30 s off). DNA fragments were end-repaired and dA-tailed using NEBNext kits and a double stranded trP1 adapter was ligated to the resulting DNA fragments. trP1 adapter ligation was confirmed by PCR using primers complimentary to the trP1 adapter. Free 3′-OH groups were blocked with Terminal Transferase (NEB) and either dideoxyATP (ddATP) or ddGTP. Samples were then treated with T4 endonuclease V (i.e. T4 PDG, NEB) and AP endonuclease APE1 (NEB) to cleave CPD lesions and cleave the resulting abasic site, respectively. 5′ phosphate groups were then removed using shrimp alkaline phosphatase (Affymetrix), and DNA was subsequently denatured at 95°C for 5 min and snap-cooled on ice.

A second double stranded adapter (A adapter) was then ligated to the 3′-OH created immediately upstream of the cleaved UV lesion using the NEBNext Quick Ligation Module. Second adapter ligation was confirmed by PCR using an unlabeled primer complementary to the trP1 adapter and a Cy3-labeled primer complementary to the A adapter (A primer). Each A adapter contains a unique barcode that allows for the creation of different libraries to be pooled and analyzed through multiplexed DNA sequencing techniques, as well as a 5′ biotin-label on one of the strands. Ligated DNA with the 5′biotin label was purified using Streptavidin beads (Thermo Fisher Scientific), the non-biotinylated DNA strand was released using 0.15M NaOH, and then used as a template for second strand synthesis using the A primer. Libraries were PCR amplified for 5–6 cycles and were generally combined at equal volumes to be submitted for Ion Proton sequencing (Life Technologies). Sequencing reads were aligned to the SacCer3 yeast genome via Bowtie2 software [42].

### CPD-seq data analysis

CPD-seq data analysis was performed as previously described [20, 22, 31]. Briefly, sequencing reads were separated based on the A adapter barcode sequences, and barcode sequences were trimmed. Sequences were then aligned to the *Saccharomyces cerevisiae* genome (saccer3) using Bowtie2 software [42], and converted to BED files using SAMtools [43] and BEDTools [44]. The CPD lesion site was determined by extracting the dinucleotide sequences immediately upstream of the 5′ end of the sequencing read, on the opposite strand using custom Perl scripts [22, 38]. Non-dipyrimidine associated reads were filtered from further analysis, and the resulting BED files were split into separate files of CPD-seq reads for the plus and minus strands. The resulting BED files were then sorted and counted using IGVtools, generating WIG files. Background files were generated to give a value of zero to each dipyrimidine in the genome that did not map a CPD lesion. For each CPD-seq experiment, the repair time point (e.g. 30 or 60 min) was analyzed relative to the matched 0 min control to determine the fraction of unrepaired CPDs remaining. The normalized fraction of CPDs remaining was standardized using the T4 endo V-alkaline gel assays. Essentially, it was calculated using a normalization factor derived from the total fraction of unrepaired CPDs at each time point for the CPD-seq data relative to the fraction of unrepaired CPDs determined by the alkaline gel assays, following our published method [32].

The normalized fraction of unrepaired CPDs along the TS and NTS of yeast genes was determined using custom Perl scripts, as previously described [20, 21]. Yeast gene transcription start site (TSS) and transcription end site (TES; i.e. polyadenylation cleavage site) were from [45]. Gene cluster plots of the fraction of unrepaired CPDs neighboring the TSS for individual yeast genes was analyzed using custom Perl scripts, essentially as previously described [21], except 1-based CPDs (as opposed to in between CPD-seq data) were analyzed, and the normalized fraction of unrepaired CPDs was visualized using the Treeview software [46].

### Analysis of repair in nucleosomes

Analysis of CPD repair in yeast nucleosomes was performed using a previously published high-resolution map of yeast nucleosome dyad positions [47]. Strongly positioned nucleosomes in this map were defined as those having a nucleosome score of greater than 5 [22]. Custom perl scripts were used to determine the fraction of CPDs remaining after 30 and 60 min of photolyase repair relative to the initial damage time point (0 min) at each position relative to the central dyad location (position 0) of 9749 strongly positioned nucleosomes, as previously described [22].

### Transcription factor analysis

We analyzed CPD repair at published TF binding sites identified by ChIP-exo (Supplementary Data 1 from [48]), using our previously described methods [49], with the exception that binding sites in the “TF,” “Cofactor,” “bound TF motif,” and “Consolidated TF” columns were also included, in addition to the sites in the “Reference Binding Event for Promoter” column that was used previously in [49]. This change resulted in a few additional binding sites for many of the TFs. Again, only binding sites associated with a consensus motif (i.e. had “motif” in name of the site) were utilized. All TF binding sites within 1 kb of a telomere end were excluded. For Reb1, we also analyzed low occupancy control binding sites (Reb1 occupancy < 10) identified in a previous study [50], excluding Reb1 sites that overlapped with repetitive genomic regions with aberrantly high sequencing depth, such as the ribosomal DNA locus, or redundant motif instances. Custom perl scripts were used to determine the fraction of CPDs remaining compared to initial damage induction (0 min) at positions relative to each TFBS midpoint. Midpoints of some TFBS were shifted by a small offset determined from the sequence logo of the motif, so that the conserved positions of the binding site were centered near the motif midpoint.

### Canavanine mutagenesis assay

Overnight cultures of YML467 were grown in rich broth (i.e. yeast extract peptone dextrose (YPD) medium or YPD medium containing adenine (YPDA)). Cells were harvested, resuspended in water, poured into a small petri dish (Falcon #351 007), and exposed to approximately 12.5 J/m^2^ UVC radiation with the lid off. Immediately following irradiation, the cells in the plate were photoreactivated by exposure to UVA light (Spectroline EA-160) for approximately 40 min with the lid on the plate. In control experiments, cells were exposed to only UVA light (i.e. no UVC). Following this procedure, cells were collected by centrifugation, resuspended in rich media, and allowed to grow overnight in the dark at 30°C with shaking. Overnight cultures were then serially diluted in water and plated on synthetic complete (SC) media or SC media lacking arginine supplemented with 0.006% canavanine (SC-Arg + Can). To determine the frequency of canavanine resistant (Can^R^) mutants, the number of colonies on SC-Arg + Can plates was divided by the number of colonies on SC plates, multiplied by its dilution. A minimum of six replicates was performed for each experimental set.

### UV passaging assay

An overnight culture of YML480 (i.e. diploid *rad14Δ* yeast expressing dPhr6-4) was diluted 100-fold in water and spotted in an 8 × 3 array onto a yeast peptone dextrose (YPD) plate. Once dry, the plate was exposed to approximately 12.5 J/m^2^ UVC radiation with the lid off. To induce photoreactivation, the plate was then incubated 40 min under a UVA lamp (Spectroline EA-160) with the lid on, and then allowed to incubate at 30°C overnight in the dark. Cells from each spot were then harvested with toothpicks and diluted with sterile water. Each sample was then spotted onto a fresh plate as done before, and the process was repeated until a total of fifteen UVC/UVA exposures had been performed. Following the final incubation, cells were struck onto fresh plates and a single isolate from each spot was grown and harvested for genomic DNA using bead beating, phenol:chloroform:isoamyl alcohol extraction, ethanol precipitation, and RNase A treatment.

Purified genomic DNA from individual yeast isolates was analyzed by whole genome sequencing, and the resulting mutations were called using CLC Workbench, as previously described [51, 52]. Only unique mutations were retained for further analysis. Analysis of transcriptional asymmetry and trinucleotide mutation spectra were analyzed using custom Perl scripts, as previously described [51, 52].

## Results

### Genome-wide role of photoreactivation in repairing UV damage in NER-proficient yeast

**Figure 1.**
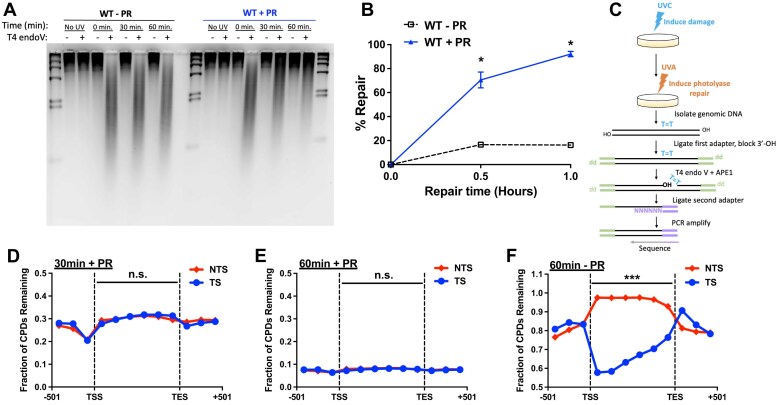
Genome-wide analysis of CPD repair in photoreactivated and NER-proficient yeast. (**A**) Representative alkaline gel of CPD repair in wild-type (WT) BY4741 cells with and without photoreaction (± PR) using UVA light. Genomic DNA was isolated at the indicated time following exposure to 100 J/m^2^ UVC light and subsequent UVA photoreactivation and treated with or without (±) T4 endonuclease V (T4 endoV) to cleave unrepaired CPD lesions. Unirradiated yeast (No UV) were included as a control (**B**) Quantification of CPD repair in WT cells based on alkaline gel analysis. The percentage of CPDs repaired is plotted as the mean ± SEM of three replicates. *P*-values were calculated using an unpaired t-test with Holm–Sidak correction for multiple hypothesis testing. *P< 0.01. (**C**) Schematic of modified CPD-seq assay. Following damage induction with 125 J/m^2^ UVC light, cells are photoreactivated for either 30 or 60 min in 2% glucose to induce repair by CPD photolyase in conjunction with NER. CPDs were mapped using the CPD-seq method with T4 endoV and APE1, as previously described [22]. (D and E) Fraction of CPDs remaining in WT cells after (**D**) 30 min and (**E**) 60 min of repair was plotted for six equally sized bins for the TS and NTS between the TSS and TES of approximately ∼5000 yeast genes. The fraction of CPDs remaining is calculated as the ratio of damage after 30 and 60 min of repair compared to the damage immediately following UV irradiation. Data for three bins comprising 167-bp upstream of the TSS and downstream of TES are also depicted. Data shown in (D and E) were normalized using the alkaline gel analysis (see panel B). n.s., not significant (*P* > 0.05) based on a paired t-test for the difference in the fraction of CPDs remaining between the TS relative to the NTS for the six coding region bins. (**F**) Same analysis as described for (D and E), except for published CPD-seq data [22] from WT cells after 1 h repair with no photoreactivation. ***P < 0.001, based on a paired t-test for the difference in the fraction of CPDs remaining between the TS relative to the NTS for the six coding region bins..

*S. cerevisiae* can repair UV-induced CPD lesions by both NER or photoreversal by CPD photolyase. While repair of CPD lesions by NER across the yeast genome has been extensively studied (e.g. [20–22, 31, 33]), the genome-wide role of CPD photolyase in UV damage repair remains unclear. In previous repair studies, yeast were typically grown in the dark to prevent photoreversal. To characterize the role of CPD photolyase in repair in yeast, we used T4 endonuclease V (T4 endo V) digestion and alkaline gel analysis [41, 53] to measure CPD repair within bulk genomic DNA (gDNA) in UVC irradiated wild-type (WT) yeast following either 30 or 60 min (min) photoreactivation with UVA light (+ PR) (Fig. 1A). As a control, similar experiments were conducted on cells kept in the dark after UVC irradiation for 30 or 60 min, in order to prevent repair by CPD photolyase (−PR). WT cells that had been photoreactivated (+PR) and could utilize both NER and CPD photolyase were able to repair ∼92% of all CPDs after 60 min (Fig. 1B), whereas cells utilizing NER alone (−PR) repaired only ∼16% of all CPDs after 60 min, a value significantly lower than that of + PR cells (*P* < 0.0001).

To characterize how CPD photolyase functions to repair CPD lesions in different genomic contexts, we used our published CPD-seq method to analyze repair of CPD lesions at single-nucleotide resolution across the yeast genome (Fig. 1C) [20, 22, 38]. Cells were exposed to UVC light to induce damage, then incubated under UVA light to induce photoreactivation for 0, 30, or 60 min, followed by CPD-seq library preparation and sequencing. Initial analysis revealed an enrichment of CPD-seq reads associated with lesion-forming dipyrimidine sequences (TT, CT, TC, and CC; see Supplementary Fig. S1A). These dipyrimidine reads decreased over the repair time course, indicative of efficient repair of CPDs by both NER and CPD photolyase.

To investigate the impact of CPD photolyase on repair of CPD lesions across all yeast genes, we analyzed the fraction of CPD lesions remaining following 30 min of UVA photoreactivation relative to the 0 min control between the TSS and TES of ∼5000 yeast genes [45]. The fraction of CPD remaining (i.e. fraction of unrepaired CPDs) calculated from our CPD-seq data was normalized using the T4 endoV alkaline gel analysis data (see Fig. 1A,B) and plotted along both the TS and non-transcribed strand (NTS) for each yeast gene, which was divided into six equally sized bins (Fig. 1D). This analysis revealed that the fraction of unrepaired CPDs was essentially the same (*P* > 0.05) along the TS and NTS of yeast genes after 30 min of photoreactivation (Fig. 1D). Analysis of the 60 min phororeactivation data showed essentially the same trend (Fig. 1E). In contrast, analysis of published CPD-seq data in non-photoreactivated (i.e. photolyase is not active) WT cells [22] revealed a much lower fraction of unrepaired CPDs on the TS of yeast genes (−PR; Fig. 1F), consistent with previous studies [22, 31].

We quantified the asymmetry in repair of the TS and NTS of yeast genes by calculating the log_2_ ratio of unrepaired CPDs on the TS relative to the NTS (Supplementary Fig. S1B), as previously described [20]. In non-photoreactivated cells (−PR), the log_2_ TS/NTS ratio was much lower than zero between the TSS and TES, reflecting fast repair of the TS due to TC-NER (Supplementary Fig. S1B). Photoreactivated cells showed a log_2_ TS/NTS ratio much closer to zero, suggesting repair of the TS and NTS occur at similar rates (Fig. S1B). Closer inspection revealed a similar fraction of unrepaired CPDs along the TS and NTS among both highly and poorly transcribed yeast genes (Supplementary Fig. S1C–H). Since, in the absence of photoreactivation, the TS of yeast genes is more rapidly repaired by the TC-NER [22, 33], these finding suggest that CPD photolyase more rapidly repairs the NTS.

### Nucleosomes inhibit repair along both the TS and NTS in photoreactivated yeast

**Figure 2.**
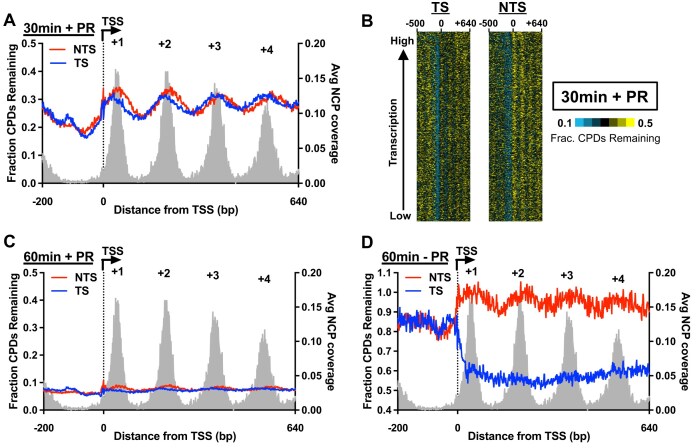
Nucleosomes inhibit photolyase repair in both the TS and NTS of yeast genes. (**A**) High-resolution analysis using CPD-seq of repair of CPDs on both the TS and NTS of ∼5200 yeast genes in WT cells photoreactivated for 30 min. The normalized fraction of CPDs remaining is calculated as the ratio of CPDs after 30 min of photoreactivation relative to the 0 min control. The average nucleosome core particle (NCP) coverage for each nucleosome is also plotted in gray [55]. (**B**) Gene plot analysis of CPD-seq data depicting the fraction of CPDs remaining after 30 min of photoreactivation in WT yeast relative to 0 min control from 500-bp upstream (−500 bp) to 640-bp downstream of the TSS of yeast genes. Repair data for the TS and NTS are plotted for genes ordered by transcriptional activity in undamaged cells [86]. (**C**) Same analysis as panel A, but examining the fraction of CPDs remaining after 60 min of photoreactivation in WT cells. (**D**) Same as panel A, but plotting the fraction of CPDs remaining after 60 min of repair in non-photoreactivated WT yeast. CPD-seq data from [22].

To analyze how genomic and chromatin features in yeast genes affect repair in photoreactivated yeast, we analyzed the fraction of CPDs remaining after 30 or 60 min photoreactivation around the TSS of ∼5200 yeast genes at single nucleotide resolution (Fig. 2). This analysis revealed a clear periodic pattern on both the NTS and TS after 30 min of repair (Fig. 2A), with peaks of unrepaired CPDs associated with positioned nucleosomes downstream of the TSS (i.e. +1 nucleosome, +2 nucleosome, etc. [54]), based on a published map of nucleosome positions [55]. A similar repair pattern was observed when the normalized fraction of CPDs remaining on the TS and NTS was plotted for genes across the genome, sorted by transcription frequency (Fig. 2B). Inspection of the resulting cluster display revealed low levels of unrepaired CPDs in the nucleosome-free region immediately upstream of the TSS and peaks of unrepaired CPDs associated with positioned nucleosomes downstream of the TSS (Fig. 2B). Analysis of the fraction of CPDs remaining within the + 1, +2, and + 3 nucleosomes downstream of the TSS confirmed that CPD repair in photoreactivated WT cells was slower near the central nucleosome dyad (Supplementary Fig. S2A). The fraction of CPDs remaining on either strand is significantly decreased after 60 min of repair (Fig. 2C), suggesting the combined activity of CPD photolyase and NER are sufficient to repair nearly all CPD lesions, even in positioned nucleosomes (Supplementary Fig. S2B). In non-photoreactivated cells, the fraction of unrepaired CPDs after 60 min is also elevated in positioned nucleosomes downstream of the TSS for the NTS of yeast genes, but not for the TS (Fig. 2D). Instead, there are much lower levels of unrepaired CPDs throughout the TS due to fast repair by the TC-NER pathway, consistent with previous reports [21, 22].

Closer inspection of the 30 min photoreactivated CPD-seq data revealed that repair inhibition in nucleosomes was asymmetric (Fig. 2A and Supplementary Fig. S2A). The peak of unrepaired CPDs in the NTS was generally on the TSS-distal side of the nucleosome dyad, while the peak of unrepaired CPDs in the TS was generally on the TSS-proximal side of the nucleosome dyad (Fig. 2A and Supplementary Fig. S2A). These findings are consistent with previous results indicating that NER causes asymmetric repair of CPD lesions in nucleosomes, with more unrepaired CPDs in the NTS in the TSS-distal side of nucleosomes in WT cells [21]. However, our data indicate that in photoreactivated cells, the TS shows the opposite pattern of repair asymmetry, with slower repair on the TSS-proximal side of nucleosomes (Supplementary Fig. S2A). Taken together, these findings indicate that in photoreactivated WT yeast, nucleosomes inhibit CPD repair in an asymmetric manner, with opposite patterns of repair asymmetry for each DNA strand.

### Role of CPD photolyase in the repair of UV damage in NER-deficient cells

The data so far indicate that photoreactivation promotes more efficient repair of CPD lesions, particularly along the NTS of yeast genes and in non-nucleosomal DNA. To directly characterize the role of CPD photolyase in repair, we analyzed repair in *rad14*Δ mutant yeast, which are NER-deficient [56]. In the absence of photoreactivation (- PR), *rad14Δ* cells are very sensitive to even a small dose of UVC light (∼6 J/m^2^, see Supplementary Fig. S3A). This UV sensitivity is partially rescued upon exposure to photoreactivating UVA light in *rad14Δ* cells (Supplementary Fig. S3A), but not in *rad14Δphr1*Δ mutants lacking CPD photolyase (i.e. *PHR1*).

**Figure 3.**
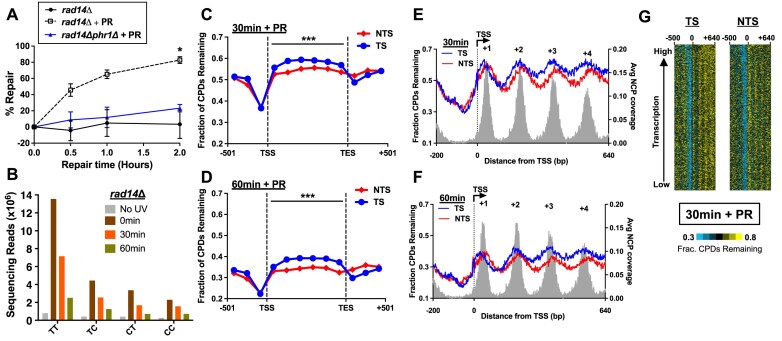
Repair of CPDs by photolyase is inhibited along the TS of yeast genes. (**A**) Quantification of CPD repair in yeast cells deficient in NER (*rad14*Δ) and/or photolyase (*phr1*Δ) using alkaline gel analysis (example gel shown in Supplementary Fig. S3B) for the indicated times of photoreactivation (+PR). The percentage of CPDs repaired is plotted as the mean ± SEM for three replicates each for *rad14*Δ +PR and *rad14*Δ (no PR) and four replicates for *rad14*Δ*phr1*Δ +PR. *P*-values were calculated using an unpaired t-test with Holm–Sidak correction for multiple hypothesis testing. *P< 0.05. (**B**) Number of CPD-seq reads associated with CPD lesions at dipyrimidine sequences in UV-irradiated *rad14Δ* cells following the indicated times of photoreactivation. (C and D) The normalized fraction of CPDs remaining in NER-deficient *rad14*Δ cells after (**C**) 30 min and (**D**) 60 min of photoreactivation was plotted for the TS and NTS for six equally sized bins between the published TSS and TES of ∼5000 yeast genes [45]. The fraction of CPDs remaining is calculated as the normalized ratio of CPD-seq reads after 30 or 60 min of photoreactivation relative to the 0 min control, normalized using the alkaline gel data (see panel A). ***P < 0.001, based on a paired t-test for the difference in the fraction of CPDs remaining between the TS relative to the NTS for the six coding region bins. (E and F) High-resolution analysis of photolyase repair after (**E**) 30 min and (**F**) 60 min of photoreactivation near the TSS of ∼5200 yeast genes in NER-deficient *rad14Δ* cells. (**G**) Gene plot analysis of CPDs remaining after 30 min of photoreactivation in NER-deficient *rad14Δ* cells along the TS and NTS of genes across the yeast genome, sorted by transcriptional activity (data from [86]).

Analysis of CPD repair in yeast by T4 endonuclease V alkaline gel analysis indicated that there was essentially no repair in *rad14*Δ cells in the absence of photoreactivating light (Supplementary Fig. S3B, quantified in Fig. 3A). However, exposure to photoreactivating UVA light promoted efficient removal of CPDs in *rad14*Δ yeast, with ∼65% of CPDs repaired by 60 min and ∼83% repaired at 2 h (Fig. 3A). Repair of CPDs in photoreactivated *rad14*Δ cells, however, was slower than photoreactivated WT cells at both the 30 and 60 min time points (compare Figs. 1B and 3A), presumably due to the absence of NER activity in these cells. Finally, *rad14Δphr1*Δ mutants lacking CPD photolyase showed little if any repair after photoreactivation (Fig. 3A), confirming that the observed repair in photoreactivated *rad14*Δ yeast is due to CPD photolyase. These results confirm that yeast photolyase can promote efficient repair of CPDs in NER-deficient yeast.

### Repair by CPD photolyase is inhibited in the TS of genes and in nucleosomes

To characterize how photolyase specifically repairs CPDs in different chromatin and genomic contexts, we used CPD-seq to measure repair of UV damage in NER-deficient *rad14*Δ cells following 0, 30, or 60 min of photoreactivation with UVA light (Fig. 1C). Analysis of the resulting CPD-seq data confirmed enrichment of sequencing reads associated with CPD-forming dipyrimidine sequences (TT, TC, CT, CC) immediately following UV damage induction (0 min) compared to the unexposed control (No UV; Fig. 3B and Supplementary Fig. S4A), which subsequently decreased at later time points (i.e. 30 and 60 min) due to repair by CPD photolyase.

Analysis of photolyase repair in yeast genes revealed faster repair of the NTS relative to TS (Fig. 3C and D). The fraction of CPDs remaining after 30 or 60 min photoreactivation in *rad14*Δ mutant cells relative to the initial 0 min control was normalized using the T4 endoV alkaline gel data (Fig. 3A) and plotted across ∼5000 yeast genes. This analysis revealed that the fraction of unrepaired CPDs after 30 or 60 min along the TS was significantly elevated relative to the NTS at each of the six equally-sized bins between the TSS and TES of yeast genes (*P* < 0.001), although the difference between the two strands was smallest in the first bin immediately downstream of the TSS (Fig. 3C and D). Photolyase repair of the TS was also slower in the transcribed regions of genes than in neighboring intergenic DNA. The fraction of CPDs remaining was similar for the TS and NTS in neighboring intergenic DNA and was lowest for both strands for the bin immediately upstream of the TSS (Fig. 3C and D).

A possible explanation for these data is that persistent RNA Polymerase II (Pol II) stalling at CPD lesions on the TS could inhibit photolyase repair, consistent with the conclusions of a previous report [18]. To test this hypothesis, we stratified the set of ∼5000 yeast genes into high transcribed (>10 mRNA per h) and low transcribed (<1 mRNA per hr) cohorts. At both 30 and 60 min, the fraction of unrepaired CPDs was significantly elevated on the NTS relative to TS for both the high expressed and low expressed gene cohorts (Supplementary Fig. S4B–E). Direct comparison of repair asymmetry revealed positive log_2_ TS/NTS values across the transcribed region of yeast genes (Supplementary Fig. S4F and G), consistent with more unrepaired CPDs on the TS. These repair asymmetry values were marginally higher for high expressed genes after 30 min repair, particularly near the TES of the gene (Supplementary Fig. S4F and G), potentially supporting the hypothesis that Pol II stalling at lesions inhibits photolyase repair.

High-resolution analysis of the fraction of CPDs remaining in photoreactivated *rad14*Δ cells around the TSS of ∼5200 yeast genes revealed a clear pattern in which peaks of unrepaired CPDs correlated with positioned nucleosomes (i.e. +1, +2, +3, etc.) downstream of the TSS (Fig. 3E and F). Nucleosomes inhibited photolyase repair downstream of the TSS on both DNA strands (TS and NTS), and peaks of unrepaired CPDs associated with nucleosomes were still apparent after 60 min of photoreactivation (Fig. 3F). There were relatively few unrepaired CPDs in the nucleosome free region immediately upstream of the TSS, consistent with fast repair by CPD photolyase in non-nucleosomal DNA.

The fraction of unrepaired CPDs was similar for the TS and NTS upstream of the TSS and in the +1 nucleosome immediately downstream of the TSS. However, more unrepaired CPDs were present on the TS further downstream of the TSS (i.e. +2, +3, and +4 nucleosomes; see Fig. 3E and F). Downstream of the +1 nucleosome, the fraction of unrepaired CPDs was elevated on the TS in both nucleosomes and linker DNA (Fig. 3E and F). Elevated levels of unrepaired CPDs along the TS, particularly distal to the TSS, was apparent for genes across the genome (Fig. 3G). Taken together, these findings indicate that repair of CPD lesions is inhibited in nucleosomes and along the TS of yeast genes downstream of the +1 nucleosome.

### CPD photolyase repairs nucleosomal DNA asymmetrically

**Figure 4.**
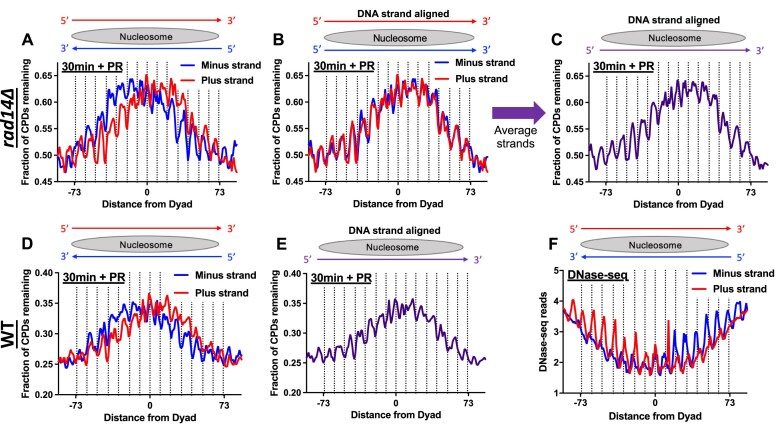
Repair of CPDs by yeast photolyase is inhibited asymmetrically in nucleosomes**. (A**) Normalized fraction of CPDs remaining within ∼10 000 strongly positioned nucleosomes after 30 min of repair (relative to 0 min control) by CPD photolyase in NER-deficient *rad14Δ* cells. Nucleosome coordinates derived from [47]. Dotted lines at positions −73 and +73 bp from the dyad center represent the predicted edges of the nucleosome core particle. Data plotted from −90 to −73 and +90 to +73 is within linker DNA. Schematic above graph represents the orientation of each DNA strand within the nucleosome core (gray oval). Dotted lines indicate minor-out rotational settings. (**B**) Same data as in (**A**), but with the minus strand of DNA oriented in the 5′ to 3′ direction. (**C**) Combined data for both strands plotted in panel B. Both DNA strands are aligned in the 5′ to 3′ orientation. (**D**) Same as panel A, except normalized fraction of CPDs remaining was analyzed after 30 min of repair in photoreactivated NER-proficient WT cells. (**E**) Combined data for both strands plotted in panel D. Both DNA strands are aligned in the 5′ to 3′ orientation. (**F**) Frequency of DNase-seq reads within ∼10 000 strongly positioned nucleosomes. DNase-seq data from [57].

Closer inspection of CPD repair in photoreactivated *rad14*Δ cells (Fig. 3E and F) revealed that the peaks in the fraction of unrepaired CPDs for the NTS and TS were offset, indicative of asymmetric repair in nucleosomes, similar to that observed in WT photoreactivated cells (Fig. 2A and Supplementary Fig. S2A). To test this hypothesis, we analyzed repair of CPD lesions by photolyase in ∼10 000 strongly positioned nucleosomes [47] across the yeast genome. After 30 min of photoreactivation in *rad14*Δ mutant cells, we observed striking repair asymmetry in strongly positioned nucleosomes (Fig. 4A). For the plus strand, unrepaired CPD lesions peaked 0–30 bases to the right of the central nucleosome dyad axis (i.e. 0 to +30). In contrast, unrepaired CPD lesions on the minus strand peaked to the left of the dyad (i.e. -30 to 0). These figures depict the CPD-seq repair data in the normal anti-parallel DNA orientation. Aligning the two DNA strands so that both the plus and minus strands run in the 5′ and 3′ orientation reveals very similar patterns of repair for the plus and minus strands (Fig. 4B). Averaging the repair data for the aligned strands revealed a peak of unrepaired CPDs on the 3′ side of the central nucleosome dyad in both DNA strands (Fig. 4C). Similar patterns were observed after 60 min photoreactivation in *rad14* mutant cells (Supplementary Fig. S5A–C), as well as in WT cells (Fig. 4D and E). Taken together, these findings indicate that the repair of UV damage by CPD photolyase is more inhibited on the 3′ side of the nucleosomal DNA on both DNA strands.

This analysis also revealed that in photoreactivated *rad14*Δ cells, there was faster repair of CPDs at positions where the minor groove of the nucleosome DNA faced outward from the histone octamer (i.e. ‘minor-out’ rotational settings; see dashed lines in Fig. 4A–C). In contrast, elevated levels of unrepaired CPDs were associated with ‘minor-in’ rotational settings, where the minor groove faces in toward the histone octamer (see peaks of unrepaired CPDs between dashed lines in Fig. 4A–C). A similar pattern of fewer unrepaired CPDs at minor-out rotational settings and high levels of unrepaired CPDs at minor-in positions was observed in strongly positioned nucleosomes after 60min of photoreactivation (Supplementary Fig. S5A–C). In general, faster repair at minor-out rotational settings was more apparent on the 5′ side of nucleosomal DNA (Fig. 4C and Supplementary Fig. S5C). There was also faster repair of CPDs at minor-out rotational settings in strongly positioned nucleosomes after 30 min of photoreactivation in WT cells (Fig. 4D and E), although the impact of rotational setting on repair was less apparent than in NER-deficient *rad14*Δ cells. After 60 min of photoreactivation in WT cells, the impact of nucleosomes on repair patterns were relatively minor (Supplementary Fig. S5D–F). In non-photoreactivated WT cells (after 60 min repair), there was no consistent pattern in unrepaired CPDs at minor-in versus minor-out rotational settings in strongly position nucleosomes (Supplementary Fig. S5G–I). Moreover, overall damage levels after 60 min were drastically lower in the photolyase active WT cells compared to cells only utilizing NER (compare Supplementary Fig. S5D–F and G–I).

Previous biochemical and genomic studies have indicated that minor-out rotation settings are more accessible to protein binding, as reflected by greater accessibility to cleavage by deoxyribonuclease (DNase) [21, 57, 58–60]. Consistent with these findings, analysis of published DNase-sequencing (DNase-seq) data in yeast [57] revealed elevated DNase-seq reads associated with cleavage of minor-out positions in the same set of ∼10 000 strongly positioned nucleosomes in yeast (Fig. 4F). Notably, DNase-seq reads were asymmetric in strongly positioned nucleosomes, being elevated on the 5′ side of the nucleosomal DNA for each DNA strand, and lower on the 3′ side (Fig. 4F), consistent with previous reports [21, 57, 60]. This pattern mirrors photolyase repair activity in nucleosomes (Fig. 4), suggesting that the faster repair of CPDs on the 5′ side of the nucleosomal DNA can be explained by greater accessibility of lesions to CPD photolyase. The correspondingly slower repair of CPDs on the 3′ side of the nucleosomal DNA is likewise due to reduced accessibility to photolyase.

### Photolyase repair is inhibited at a subset of transcription factor binding sites

**Figure 5.**
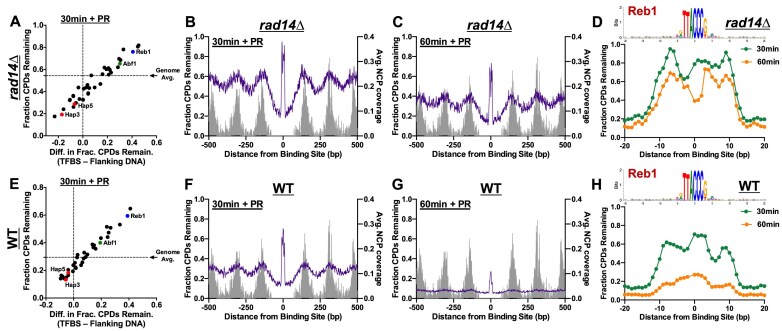
DNA binding by a subset of yeast transcription factors inhibits photolyase repair. (**A**) Plot of the fraction of CPDs remaining (*y*-axis) after 30-min photoreactivation in *rad14*Δ cells relative to the 0 min control within the core binding sites (i.e. −4 to +4 bp from the motif midpoint) of each of 42 different yeast transcription factors derived from published ChIP-exo data [48]. Horizontal dashed line indicates the overall (average) fraction of CPDs remaining across the genome. The fraction of CPDs remaining was normalized using the alkaline gel T4 endo V assay (see Materials and methods). *X*-axis plots the difference in fraction of CPDs remaining between the core binding site (i.e. −4 to +4 bp from the motif midpoint) and adjacent flanking DNA (i.e. from −100 to +100 bp relative to the motif midpoint, excluding the core binding site [−4 to +4 bp]). Binding site data for Hap3 and Hap5 TFs are indicated in red, Reb1 TF in blue, and Abf1 TF in green. Only the 42 TFs with at least 100 CPDs in the core binding site at both 0 and 30 min time points are depicted. (B and C) The fraction of CPDs remaining for positions −500 to +500 bp from the midpoint of 339 Reb1 binding sites derived from published ChIP-exo data [48] following (**B**) 30 min and (**C**) 60 min of CPD photolyase repair in *rad14Δ* cells. The fraction of CPDs remaining was normalized using the alkaline gel T4 endo V assay (see Materials and methods). The average nucleosome core particle (NCP) coverage for each nucleosome (MNase-seq data from [55]) is plotted in gray. Positions associated with fewer than 10 CPDs in either time point were not plotted. (**D**) Closer examination of CPD-seq data shown in panels B and C. Distance spans −20 and +20 bp from midpoint (position 0) of binding motif. Binding motif logo created using weblogo3 software [87]. (**E**) Same as panel A, except for after 30-min photoreactivation in NER-proficient WT cells relative to the 0 min control. (F and G) Same as panels B and C, except depicting fraction of CPDs remaining at 339 Reb1 binding sites following (**F**) 30 min and (**G**) 60 min of photoreactivation in WT cells. (**H**) Same as panel D, except showing data for NER-proficient WT cells.

Previous genome-wide repair studies have indicated that DNA binding by transcription factors (TFs) can inhibit removal of UV damage by the NER pathway [22, 26–28, 61]; however, to what extent DNA-binding by different classes of TFs affect photolyase repair remains largely unclear. To address this question, we analyzed the fraction of CPDs remaining after 30 min of photoreactivation in *rad14Δ* cells within the binding sites for 42 different TFs in yeast. Transcription factor binding site (TFBS) coordinates were derived from a published chromatin immunoprecipitation exonuclease sequencing (ChIP-exo) study in yeast [48]. Since many TFBS are located in nucleosome-free promoter regions, which our data indicate is rapidly repaired by photolyase in yeast (see Fig. 3E and F), we also plotted the difference in CPDs remaining in the TFBS relative to DNA immediately flanking the TFBS (*x*-axis of Fig. 5A). This analysis revealed that for a subset of yeast TFs, including Reb1 and Abf1, the fraction of unrepaired CPDs in the binding site was higher than the genome average (see dashed line in Fig. 5A) or that of adjacent flanking DNA. However, at other classes of TFBS, including binding sites of the Hap3/Hap5 TFs [62], there was a lower fraction of unrepaired CPDs in the binding site relative to the genome average or adjacent flanking DNA (Fig. 5A), indicating that these and a number of other classes of TFs do not inhibit repair by photolyase.

We focused our analysis on Reb1, since it strongly inhibits repair of CPDs by photolyase and because it is one the most abundant classes of TFBS in the yeast genome. Analysis of photolyase repair of CPDs in 339 Reb1 binding sites within photoreactivated *rad14Δ* cells revealed clear repair inhibition centered at the TFBS after both 30 min (Fig. 5B) and 60 min (Fig. 5C). In contrast, there were low levels of unrepaired CPDs in DNA regions immediately flanking (i.e. within 100 bp) the Reb1 binding site, likely because these regions are free of positioned nucleosomes (see gray bars in Fig. 5B and C) and therefore rapidly repaired by photolyase. Peaks of unrepaired CPDs were also observed at more distal positions from the Reb1 TFBS and were associated with positioned nucleosomes (Fig. 5B and C). Closer inspection of the data (Fig. 5D) indicated that the peak of unrepaired CPDs not only encompassed the core Reb1 binding motif (i.e. TTACCC), but also extended roughly 5 bp beyond the motif in both directions (i.e. −10 to +10 bp from the motif midpoint, see Fig. 5D). Similar analysis of a control set of Reb1 binding motifs associated with low Reb1 occupancy revealed only slight elevation in unrepaired CPDs after 30 or 60 min of photoreactivation in *rad14*Δ cells (Supplementary Fig. S6A–C), indicating that Reb1 binding is required for inhibition of photolyase repair.

Our initial analysis indicated that binding sites of the yeast TF Abf1, which are also abundant in the yeast genome, are associated with elevated levels of unrepaired CPDs in photoreactivated *rad14*Δ cells (Fig. 5A). Analysis of Abf1 binding sites revealed a similar repair pattern, in which CPD photolyase is inhibited at the center of the Abf1 binding motif, as well as in neighboring nucleosomes (Supplementary Fig. S7A-C), and this inhibition persists for the entire 60 min time course. Notably, elevated levels of unrepaired CPDs extended ∼5 bp beyond the consensus Abf1 motif in each direction (Supplementary Fig. S7C), encompassing −12 to +12 bp from the TFBS midpoint. Taken together, these data indicate that repair of CPDs by photolyase is inhibited by DNA-binding by Reb1, Abf1, and a subset of other yeast TFs.

Analysis of CPD repair in TFBS in photoreactivated WT cells revealed a similar trend (Fig. 5E), with elevated levels of unrepaired CPDs associated with Reb1, Abf1, and a subset of other TFBS. Similar to what was observed in *rad14Δ* cells, there were elevated levels of unrepaired CPDs at the center of Reb1 binding sites after 30 min of NER and CPD photolyase activity (Fig. 5F), although the magnitude of this inhibition was less than what was observed in *rad14Δ* cells (Fig. 5B). There were much lower levels of unrepaired CPDs in flanking nucleosomes, consistent with relatively efficient repair in nucleosomes by NER. Again, there was little if any repair inhibition at a control set of low occupancy Reb1 binding motifs (Supplementary Fig. S6D–F). After 60-min photoreactivation in WT cells, there were generally low levels of unrepaired CPDs in neighboring nucleosomes, with some repair inhibition persisting at Reb1 binding sites (Fig. 5G and H). Similar repair patterns in photoreactivated WT cells were observed at Abf1 binding sites (Supplementary Fig. S7D–F).

### Genome-wide analysis of UV mutations in photoreactivated NER-deficient cells

The data so far indicate that yeast photolyase rapidly repairs CPDs throughout the genome, but is inhibited in certain chromatin and genomic contexts, including the TS of most yeast genes. To determine what impact this repair inhibition has on UV mutagenesis, we used whole genome sequencing to identify UV-induced mutations in NER-deficient (*rad14*Δ) cells that were photoreactivated. Because *rad14*Δ cells are highly sensitive to UV, even in the presence of photoreactivating UVA light (Supplementary Fig. S8), we integrated the gene encoding the *Drosophila melanogaster* 6–4PP photolyase (dPhr6-4), so that photoreactivation would repair both of the major UV-induced photoproducts (i.e. CPDs and 6-4PPs). Transgenic *rad14*Δ cells containing both endogenous CPD photolyase and the dPhr6-4 were resistant to a low dose of UVC light (12.5 J/m^2^) when subsequently photoreactivated with UVA, but were highly sensitive in the absence of photoreactivation (Supplementary Fig. S8A). Moreover, *rad14*Δ cells lacking either endogenous CPD photolyase (*phr1*Δ) or the dPhr6-4 transgene were highly sensitive to this UVC dose (Supplementary Fig. S8A). Notably, exposure of the *rad14*Δ *PHR1* dPhr6-4 strain containing both photolyases to 12.5 J/m^2^ of UVC light followed by 40 min of UVA photoreactivation still resulted in a nearly 50-fold induction of mutations in the *CAN1* reporter gene relative to cells not exposed to UVC light (Supplementary Fig. S8B).

**Figure 6.**
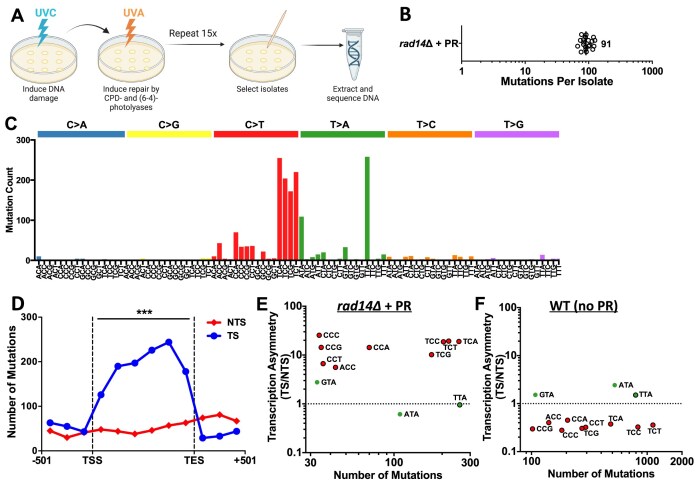
UV-induced mutations are elevated on the TS of yeast genes in NER-deficient photoreactivated yeast. (**A**) Schematic detailing UV passaging assay to induce mutations in NER-deficient (*rad14*Δ) diploid yeast containing both endogenous CPD photolyase and a dPhr6-4 transgene. Yeast cells were exposed to 12.5 J/m^2^ of UVC light, after which cells were incubated under 40 min of photoreactivating UVA light to induce repair by both CPD and 6–4PP photolyases and allowed to regrow for 1–2 days in the dark on YPD plates. This process was repeated 15 times, and individual clonal isolates were selected for whole genome sequencing. Schematic created using BioRender. (**B**) Number of mutations per isolate identified by whole genome sequencing of *rad14*Δ isolates (with endogenous CPD photolyase and dPhr6-4 photolyase) that had been photoreactivated (+PR) using the UV passaging assay shown in panel A. (**C**) Mutation classes identified in UV-irradiated and photoreactivated *rad14*Δ cells with endogenous CPD photolyase and d6-4PP photolyase transgene. Count of each class of mutations (i.e. C > A, C > G, C > T, etc.) in different trinucleotide sequence context. (**D**) Distribution of UV mutations in *rad14*Δ + PR cells on the TS and NTS within ∼5000 yeast genes. Each gene was divided into six equally sized bins between the TSS and TES and the total count of mutations in each bin and on each strand is plotted. Data for three bins, each comprising 167 bp of DNA, upstream of the TSS and downstream of TES are also depicted. ***P < 0.001, based on a paired t-test for the difference in the fraction of CPDs remaining between the TS relative to the NTS for the six coding region bins. (**E**) Strand asymmetry of UV-induced mutations in *rad14*Δ cells (with endogenous CPD photolyase and d6-4PP photolyase transgene) + PR. Normalized ratio of mutations for each mutation class and trinucleotide sequence context on the TS relative to the NTS (i.e. transcriptional asymmetry), normalized by the distribution of each trinucleotide sequence context on both DNA strands. C > T mutations are indicated in red circles and T > A mutations are indicated by green circles. No other trinucleotide mutation classes had at least 30 mutations. Black outline indicates mutation occurs in a dipyrimidine sequence context. (**F**) Same as panel E, except showing transcriptional asymmetry for the same mutation classes and trinucleotide contexts for non-photoreactivated WT cells.

To accumulate UV-induced mutations in photoreactivated cells, we generated diploid yeast containing homozygous deletion of the *RAD14* gene and a single copy of the dPhr6-4 transgene. Independent isolates of these cells were subjected to 15 doses of UVC (12.5 J/m^2^) immediately followed by photoreactivation with 40 min of UVA light after each UVC dose (Fig. 6A). Yeast isolates were allowed to regrow 1–2 days after each UVC/photoreactivation treatment. Genomic DNA was isolated from individual clones derived from 20 independent yeast isolates and subjected to a whole genome sequencing, as previously described [51, 52], in order to identify the resulting UV-induced mutations.

Bioinformatics analysis of the resulting whole genome sequencing data indicated that photoreactivation of *rad14*Δ mutant cells with the *Drosophila* dPhr6-4 transgene contained a median of 91 mutations per isolate (Fig. 6B). Analysis of the resulting mutation spectrum (Fig. 6C) revealed enrichment of C > T mutations in CPD-forming dipyrimidine sequences and T > A substitutions primarily in NTA sequence contexts (i.e. ATA, CTA, GTA, and TTA). Our previous studies indicate that this latter class of mutations are likely caused by atypical thymine-adenine (TA) photoproducts [49, 51, 52]. The relative abundance of T > A substitutions in photoreactivated *rad14*Δ cells is consistent with the model that TA photoproducts cannot be repaired by either photolyase. In contrast, C > T mutations in dipyrimidine sequences match the canonical UV mutation signature, and have been primarily attributed to mutagenic bypass of CPDs [63, 64].

Analysis of the distribution of these UV-induced mutations across the transcribed regions of ∼5000 yeast genes revealed significant enrichment of mutations on the TS (Fig. 6D). There were ∼4-fold more mutations on the TS than the NTS of yeast genes, an enrichment that was apparent even after normalizing for the sequence context of the DNA strands (Supplementary Fig. S8C). Mutations were also elevated on the TS relative to flanking intergenic DNA (Fig. 6D and Supplementary Fig. S8C). Transcriptional asymmetry analysis revealed that all C > T mutation classes occurred at least 5-fold more frequently on the TS than the NTS in photoreactivated *rad14Δ* cells (Fig. 6E), whereas these same mutation classes occurred less frequently on the TS in WT cells (Fig. 6F), due to rapid repair by the TC-NER pathway [52]. These findings suggest that inhibition of photolyase repair by Pol II stalling at UV photoproducts (i.e. CPDs and 6-4PPs) results in elevated mutation rates along the TS of yeast genes.

## Discussion

**Figure 7.**
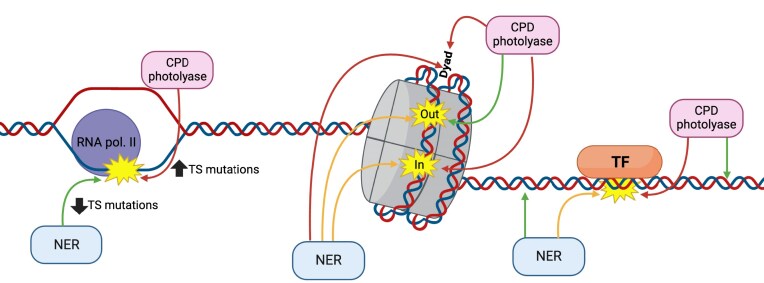
Model of CPD repair by photolyase and NER in different chromatin contexts across the yeast genome. CPD photolyase repair is significantly inhibited on the TS of yeast genes and repairs slowly within nucleosomes and some TFBS but demonstrates efficient repair elsewhere. Green arrows indicate fast repair, yellow arrows indicate intermediate rates of repair, and red arrows indicate slow repair. Created in BioRender. Bohm, K. (2025) https://BioRender.com/s9obek5.

Photolyase enzymes play a critical role in repairing UV damage in many species, including bacteria, yeast, and other eukaryotes. Here, we report the first genome-wide map of CPD repair by photolyase. Our data indicate that yeast photolyase rapidly repairs CPDs, particularly in nucleosome free regions (NFR), linker DNA, and the NTS of yeast genes, but photolyase activity is impaired in nucleosomes, certain classes of TFBS, and along the TS of most yeast genes (Fig. 7). Our data also suggest that in such slow-repairing regions of the genome, NER plays a critical role in repairing CPDs to prevent mutations. In support of this model, we show that in photolyase-active, NER-deficient cells, UV mutations are significantly elevated along the slow-repairing TS of yeast genes. These findings reveal the ‘division of labor’ of NER and photolyase in repairing UV damage across a eukaryotic genome and highlight the importance of TC-NER in preventing UV mutagenesis in photoreactivated cells.

Our data indicate that photolyase repair is inhibited in strongly positioned nucleosomes throughout the yeast genome, with repair inhibition being highest near the central dyad axis and lowest at the nucleosomal DNA exit/entry regions and in adjacent linker DNA. Within nucleosomes, photolyase repair was generally faster at minor-out rotational settings, where the DNA minor groove faces away from the histone octamer, and slower at minor-in rotational settings (Fig. 7). These findings are consistent with previous studies of photolyase repair at individual yeast genes and in nucleosomes reconstituted *in vitro* [17, 34, 36, 37, 65], and likely reflect differences in DNA accessibility to photolyase in nucleosomes. Our data also indicate that photolyase repair inhibition in nucleosomes is asymmetric, with lesions on the 3′ side of each nucleosomal DNA strand generally being repaired slower than lesions at equivalent positions on the 5′ side of the dyad. A very similar asymmetry was previously detected for repair of CPDs in nucleosomes by the NER pathway [21]. In both cases, this repair asymmetry likely arises from differential accessibility of the 5′ and 3′ sides of the nucleosomal DNA, a consequence of the left-handed wrapping of the DNA superhelix around the histone octamer [21, 57, 59]. This model is supported by our analysis of published DNase-seq data in yeast [57], which revealed elevated DNase I activity on the 5′ side of the nucleosomal DNA for both DNA strands, consistent with previous results [21, 57]. Analysis of CPD repair in photoreactivated and NER-proficient (i.e. WT) cells indicates that nearly all CPDs are repaired in nucleosomes by 1 h, suggesting that NER promotes repair of regions of nucleosomal DNA that are less accessible to photolyase (Fig. 7).

We also observed that repair by CPD photolyase is inhibited at specific classes of TFBS, including binding sites of Reb1, Abf1, and a handful of other yeast TFs. In contrast, other classes of TFBS, including binding sites of the Hap2/Hap3/Hap5 complex [62], showed essentially no inhibition of photolyase repair. One possible explanation is that these classes of TFs (e.g. Hap3/Hap5, etc.) are unable to remain bound to CPD-containing binding sites and thus do not interfere with photolyase repair. In contrast, Reb1, Abf1, and potentially other yeast TFs may have sufficient binding affinity to remain bound to CPD-containing sites, which can explain why subsequent photolyase repair is inhibited. This model is consistent with previous reports indicating that the binding affinity and specificity of many TFs is significantly reduced and/or altered when UV damage is present in the binding site [66–68]. However, certain classes of TFs (e.g. TBP, CTCF, etc.) are able to bind to CPD-containing binding sites and can inhibit subsequent damage recognition and/or repair, either by a model repair enzyme [26], NER-competent *Xenopus* oocyte nuclear extracts [69], UV-damaged DNA binding protein (UV-DDB) [68], or CPD photolyase [16]. Detailed analysis of photolyase repair at Reb1 and Abf1 binding sites indicates that repair inhibition in these TFBS is greater than inhibition in neighboring nucleosomes, and extends ∼5 bp in both directions beyond the binding site consensus motif. This latter observation can be potentially explained by structural studies indicating that CPD photolyase binds the phosphate backbone of multiple bases flanking the CPD (reviewed in [6]). Hence, Reb1 or Abf1 binding adjacent to a CPD may interfere with these extended photolyase-DNA backbone interactions and thereby inhibit repair by preventing CPD recognition. In NER-proficient cells, there were much fewer unrepaired CPDs after 1 h of photoreactivation at Reb1 and Abf1 binding sites, consistent with the model that NER promotes repair of TFBS that are refractory to photolyase (Fig. 7).

Previous reports have suggested that photolyase enzymes may also promote repair of UV damage in the absence of photoreactivating light by stimulating NER [70–73]. In yeast, this conclusion is primarily based on a study indicating that a photolyase mutant (*phr1*Δ) exacerbates the UV sensitivity of yeast cells defective in post-replication repair (*rad18*Δ) incubated in the dark, but not NER-deficient cells (*rad2*Δ) [70]. Our UV sensitivity data indicates that *phr1*Δ cells incubated in the dark do not show elevated sensitivity to either a low dose of UV irradiation (Supplementary Fig. S3A) or a significantly higher UV dose (Supplementary Fig. S9). These findings are consistent with previous reports indicating that *phr1*Δ mutant yeast in an otherwise WT genetic background (i.e. *RAD18* WT) show roughly similar UV sensitivity when incubated in the dark as WT cells over a wide range of UV doses [74, 75]. Consistent with these findings, XR-seq analysis of UV damage repair in *E. coli* cells lacking photolyase did not observe a defect in NER throughout the genome, nor along the TS and NTS of genes, in *phr*- cells incubated in the dark [76]. These findings do not rule out the possibility that photolyase may contribute to NER activity in yeast (or *E. coli*), but this contribution may only be apparent in cells otherwise defective in other repair pathways [76]. Future studies will be needed to test this hypothesis.

In mammalian cells and non-photoreactivated yeast, CPDs on the TS of genes across the genome are repaired more rapidly than CPDs on the NTS, due to rapid repair by the TC-NER pathway [22, 25, 31, 33, 77]. In human skin cancers, this results in lower rates of somatic mutations on the TS of human genes [78]. However, our CPD-seq data indicate that in photoreactivated yeast that are NER-proficient, both strands (i.e. TS and NTS) of yeast genes are repaired with similar efficiency. Our data also indicate that photolyase more rapidly repairs CPDs on the NTS of yeast genes than the TS, thereby compensating for slower NER of CPDs on the NTS. These findings are consistent with previous reports indicating that photolyase repair in yeast is inhibited along the TS of the *GAL10* gene in a transcription-dependent manner, as well as the *HIS3* and *URA3* genes [18, 34]. The simplest explanation for these findings is that RNA Pol II stalling at a CPD prevents photolyase from accessing the CPD and repairing it (Fig. 7). Consistent with this model, a previous study showed that Pol II stalling at a site-specific CPD lesion inhibits photolyase repair *in vitro* [79]. It will be of interest in future studies to characterize cellular proteins that affect photolyase repair inhibition along the TS of yeast genes, potentially due to their role in regulating Pol II stalling and/or eviction.

Whole genome sequencing of UV-induced mutations in photoreactivated yeast deficient in NER (*rad14*Δ) indicate that slow repair of the TS by photolyase translates to increased UV mutation rates along the TS of yeast genes. Across the genome, 64% of the UV-induced mutations occurred on the TS of yeast genes, while only 16% of mutations occurred on the NTS. This 4-fold difference in mutation rate between the two DNA strands is likely an underestimate, since 23% of all single base substitutions were T > A substitutions at NTA sequence contexts (i.e. NTA > NAA mutations), a mutation signature previously linked to atypical thymine-adenine (TA) photoproducts [51, 52, 80, 81]. If this class of mutations is excluded (i.e. since TA photoproducts are unlikely to be repaired by either CPD or 6-4PP photolyases), then there are ∼8-fold more mutations on the TS relative to the NTS of yeast genes. This difference in strand-specific mutation frequency in yeast genes is greater than the difference in strand-specific repair by CPD photolyase (see Fig. 3 and Supplementary Fig. S4). This may simply be a consequence of ongoing inhibition of photolyase repair along the TS causing a cumulatively greater difference in mutation frequency. Alternatively, it is possible that the exogenous 6-4PP photolyase expressed in the *rad14*Δ strain may be more strongly inhibited by Pol II stalling at a 6-4PP, or that Pol II stalling at a CPD may render it more mutagenic, for example, by stimulating the rate of deamination of cytosine-containing CPDs to uracil. The rate of cytosine deamination in a CPD is orders of magnitude higher than undamaged DNA, particularly if a CPD is resident in single-stranded DNA [82, 83]. Hence, Pol II stalling at a cytosine-containing CPD may not only protect it from photolyase repair, but may also render it more susceptible to deamination by trapping it in a single-stranded state in the Pol II active site [84]. Future studies will be required to test these hypotheses.

In conclusion, our genome-wide map of photolyase repair in yeast indicates that photolyase rapidly removes CPDs across much of the genome, but has difficulty repairing damage in certain genomic and chromatin contexts, including nucleosomes, certain classes of TFBS, and the TSs of genes. In such contexts, NER plays a critical role in removing CPDs, in addition to its role in repairing other forms of UV damage, such as atypical TA photoproducts [52, 80, 85], which cannot be repaired by photolyase. Moreover, we show that mutations accumulate on the TS of yeast genes in NER-deficient yeast that are photoreactivated, highlighting the critical role of TC-NER in preventing UV-induced mutations in photoreactivated cells. Photolyase enzymes are found ubiquitously throughout nature, ranging from single-cell prokaryotes, archaea, and eukaryotes to highly complex multicellular organisms, including many plant and animal species [6, 7]. Our findings have important implications for understanding DNA repair and mutagenesis in organisms that use photolyase enzymes to survive in persistently damaging environments due to sun exposure.

## Supplementary Material

gkaf495_Supplemental_File

## Data Availability

The CPD-seq data generated in this study have been deposited in the NCBI Gene Expression Omnibus (GEO) database under accession GSE280082. The whole genome sequencing data for calling UV-induced mutations in photoreactivated rad14Δ yeast have been deposited in the NCBI Sequence Read Archive (SRA; https://www.ncbi.nlm.nih.gov/sra) under BioProject accession PRJNA1168543. Previously published CPD-seq data for WT cells after 1 h of repair in the dark (no photoreactivation) is available under GEO accession GSE79977. Custom software code used for this analysis can be found through Zenodo at https://doi.org/10.5281/zenodo.15259785.
