# Supplementary material for: Genome-wide maps of UV damage repair and mutation suppression by CPD photolyase
Source: Nucleic Acids Res. 2025 Jun 11;53(11):gkaf495. doi: 10.1093/nar/gkaf495 (PMC12153352; doi:10.1093/nar/gkaf495)
Supplement: gkaf495_Supplemental_File [file gkaf495_supplemental_file.pdf]

Supplemental Materials

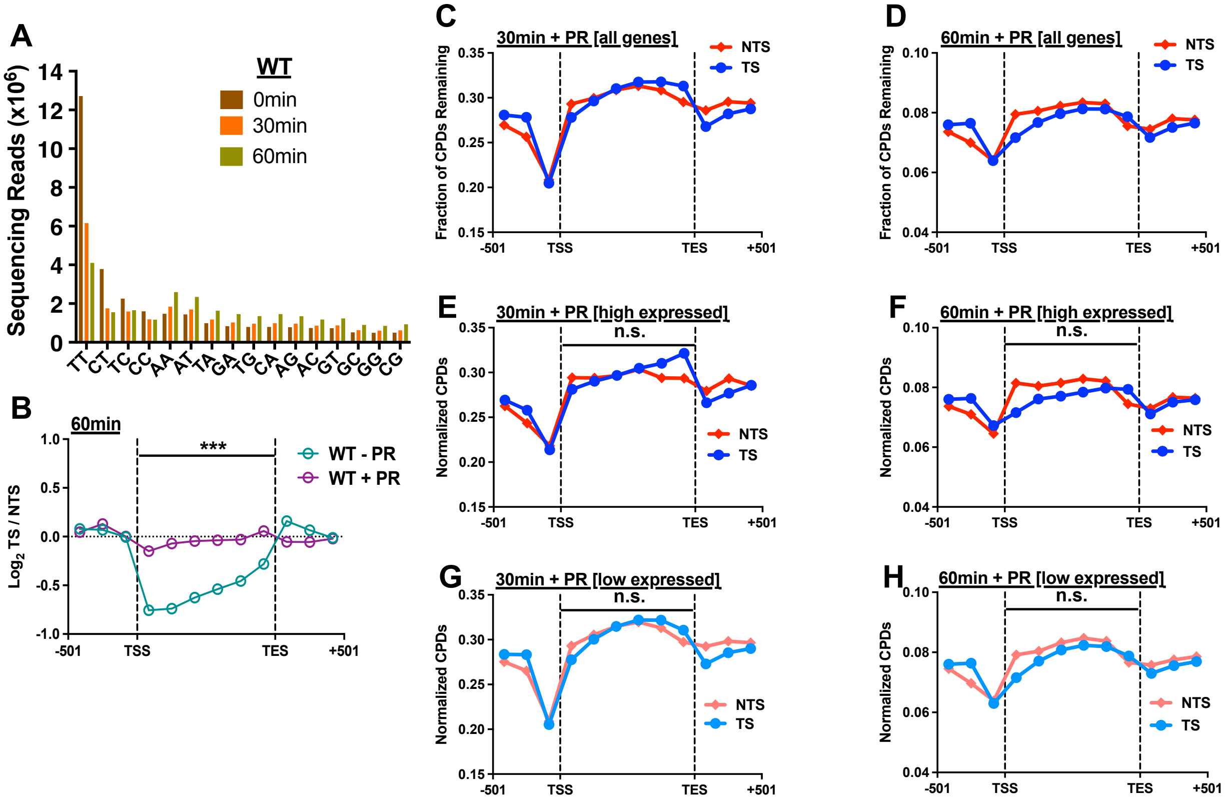

Figure S1: (A) Counts of CPD-seq reads associated with putative lesions at the indicated dinucleotide sequences in UV-irradiated WT cells following the indicated times (e.g., 0, 30, and 60 minutes (min)) of photoreactivation (PR) with UVA light. (B) Plot showing the transcriptional asymmetry in repair of CPDs, which is quantified using the $\log_2$ ratio of CPDs remaining on the TS versus the NTS for ~5000 yeast genes after 60 minutes of repair in WT cells (relative to the 0min control) with and without photoreactivation (+/- PR). Values near zero indicate low transcriptional asymmetry between the TS and NTS, whereas negative values indicate large transcriptional asymmetry, with less damage remaining on the TS compared to the NTS. (C-D) Same as Figure 1 E-F, with the Y-axis zoomed in to show detail in the repair patterns on each DNA strand. (E-H) Gene plot analysis of the normalized fraction of unrepaired CPDs at

the indicated time points relative to the 0min control within (E-F) highly transcribed genes (>10 mRNAs per hour) and (G-H) lowly transcribed genes (<1 mRNAs per hour). Each gene was divided into six equally sized bins between the transcription start site (TSS) and transcription end site (TES). Three additional bins of 167 bp in size were analyzed upstream of the TSS and downstream of the TES. Transcriptional frequencies are derived from (1). n.s., not significant ($P > 0.05$) based on a paired t-test for the difference in the fraction of CPDs remaining between the TS relative to the NTS for the six coding region bins.

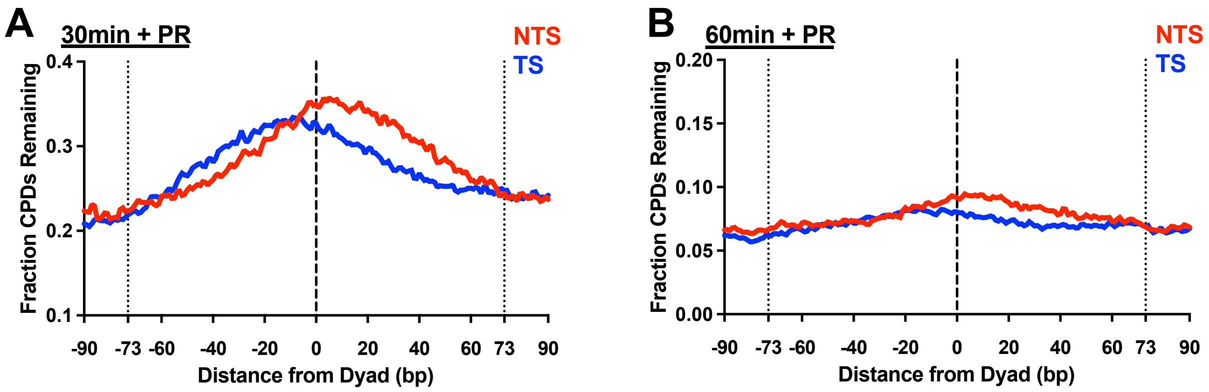

Figure S2: (A-B) Normalized fraction of CPDs remaining after (A) 30min and (B) 60min of photoreactivation relative to the 0 min control in WT cells within the first three nucleosomes immediately downstream of the transcription start site (TSS) of ~5200 yeast genes (i.e., +1 nucleosome, +2 nucleosome, and +3 nucleosome). The fraction of CPDs remaining was normalized using the T4 endoV alkaline gel data and depicted separately for the non-transcribed strand (NTS) and transcribed strand (TS) of yeast genes. Nucleosome positions obtained from (2).

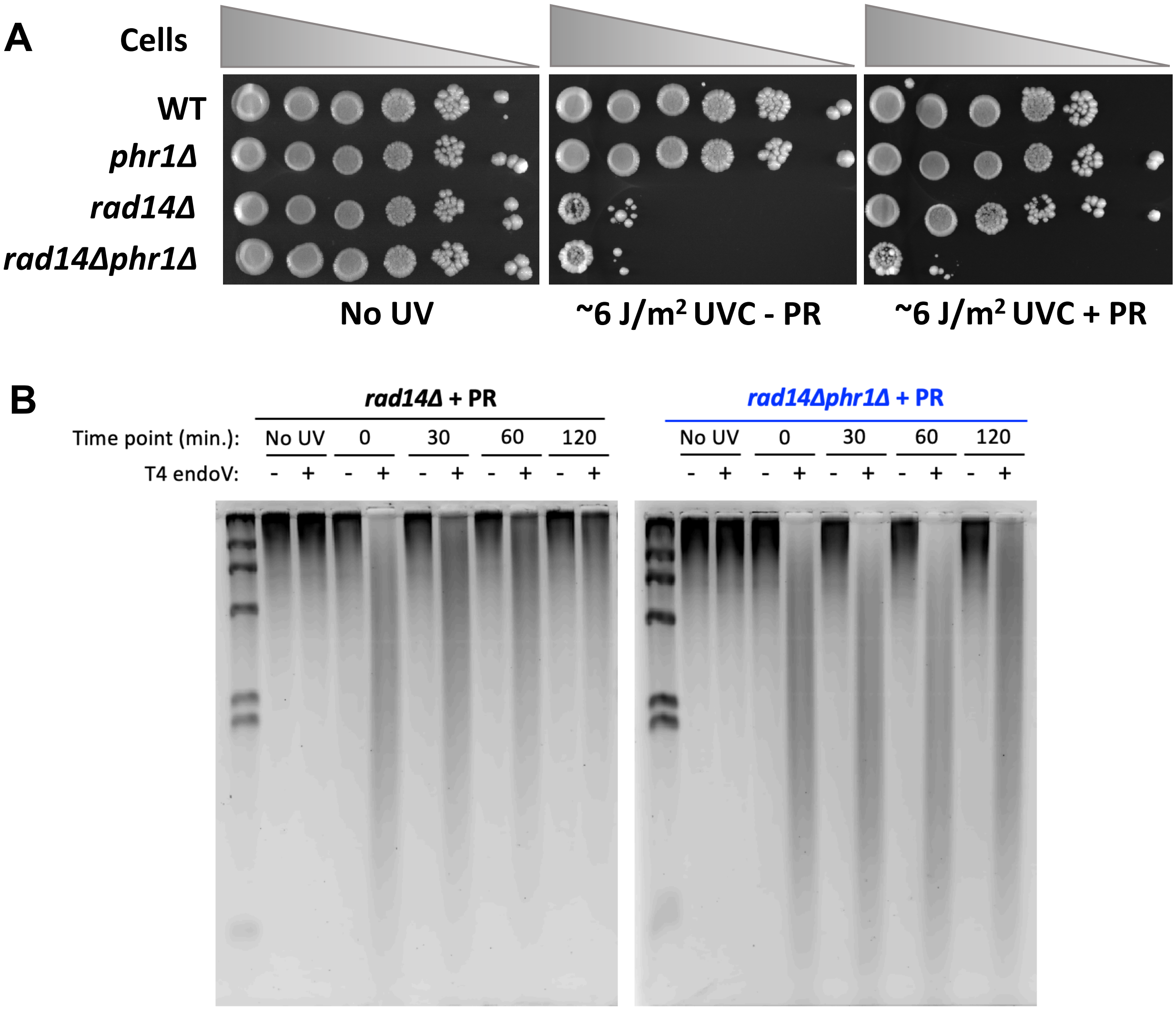

Figure S3: (A) UV sensitivity of WT yeast and yeast mutants deficient in photolyase (*phr1*Δ), NER (*rad14*Δ), or NER and CPD photolyase (*rad14*Δ*phr1*Δ). Photoreactivated cells (+PR, right panel) were incubated under 365nm photoreactivating light for 40 minutes post UVC exposure. Following treatments, all plates were incubated in darkness at 30°C and photographed. (B) Representative alkaline gel of CPD repair in *rad14*Δ and *rad14*Δ*phr1*Δ cells with photoreactivation (+PR). Genomic DNA was isolated at the indicated times (time point) following exposure to 100J/m^2 UVC light and treated with or without (+/-) T4 endonuclease V.

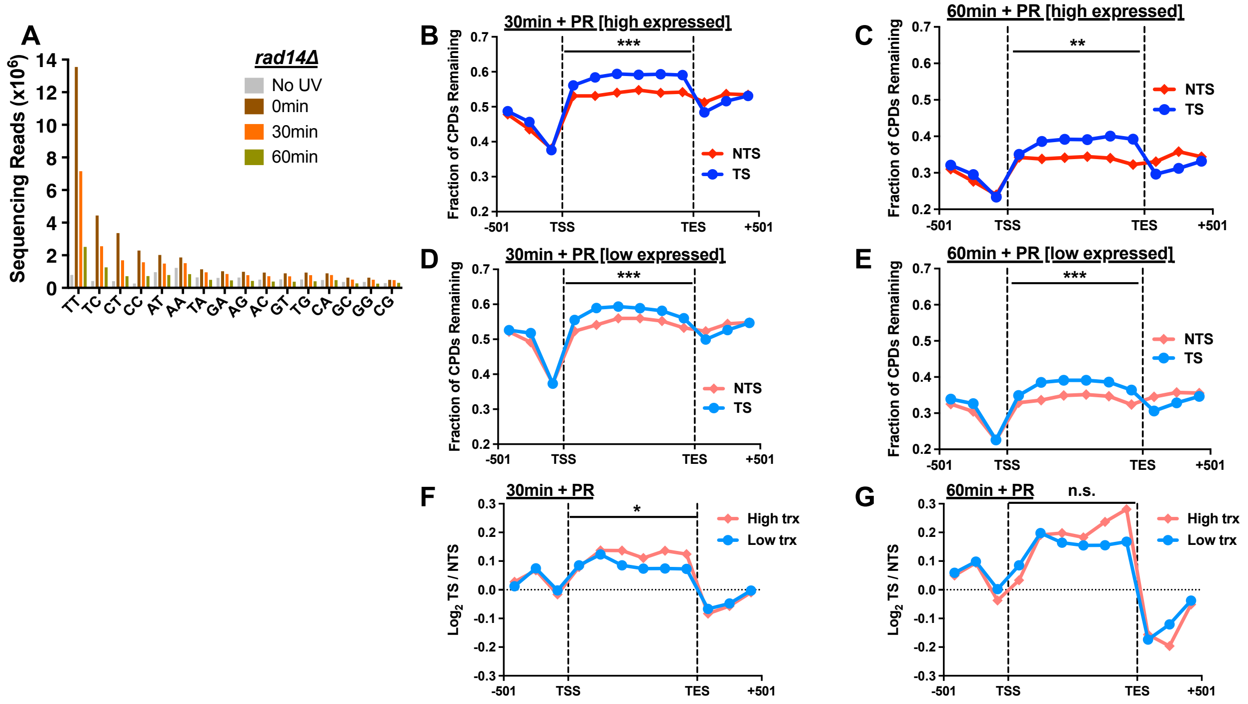

Figure S4: (A) Counts of CPD-seq reads associated with putative lesions associated with the indicated dinucleotide sequences in UV-irradiated *rad14*Δ cells following the indicated times (e.g., 0, 30, and 60 min) of photoreactivation (PR) with UVA light. (B-E) Gene plot analysis of the normalized fraction of unrepaired CPDs at the indicated time points relative to the 0min control within (B-C) highly transcribed genes (>10 mRNAs per hour) and (D-E) lowly transcribed genes (<1 mRNAs per hour). Transcriptional frequencies are derived from (1). Each gene was divided into six equally sized bins between the transcription start site (TSS) and transcription end site (TES). Three additional bins of 167 bp in size were analyzed upstream of the TSS and downstream of the TES. ***P < 0.001; **P < 0.01, based on a paired t-test for the difference in the fraction of CPDs remaining between the TS relative to the NTS for the six coding region bins. (F-G) Plot showing the transcriptional asymmetry in repair of CPDs, which is quantified using the $\log_2$ ratio of normalized CPDs remaining on the TS versus the NTS

of highly transcribed and lowly transcribed genes in *rad14*Δ cells after (F) 30min and (G) 60min of photoreactivation relative to the 0min control. Log_2 TS/NTS values near zero indicate low transcriptional asymmetry between the TS and NTS, whereas positive values indicate large transcriptional asymmetry, with more unrepaired CPDs on the TS compared to the NTS. n.s., not significant ($P > 0.05$); *$P < 0.05$, based on a paired t-test for the difference in log_2 ratio of normalized CPDs remaining on the TS versus the NTS between highly transcribed and lowly transcribed genes for the six coding region bins.

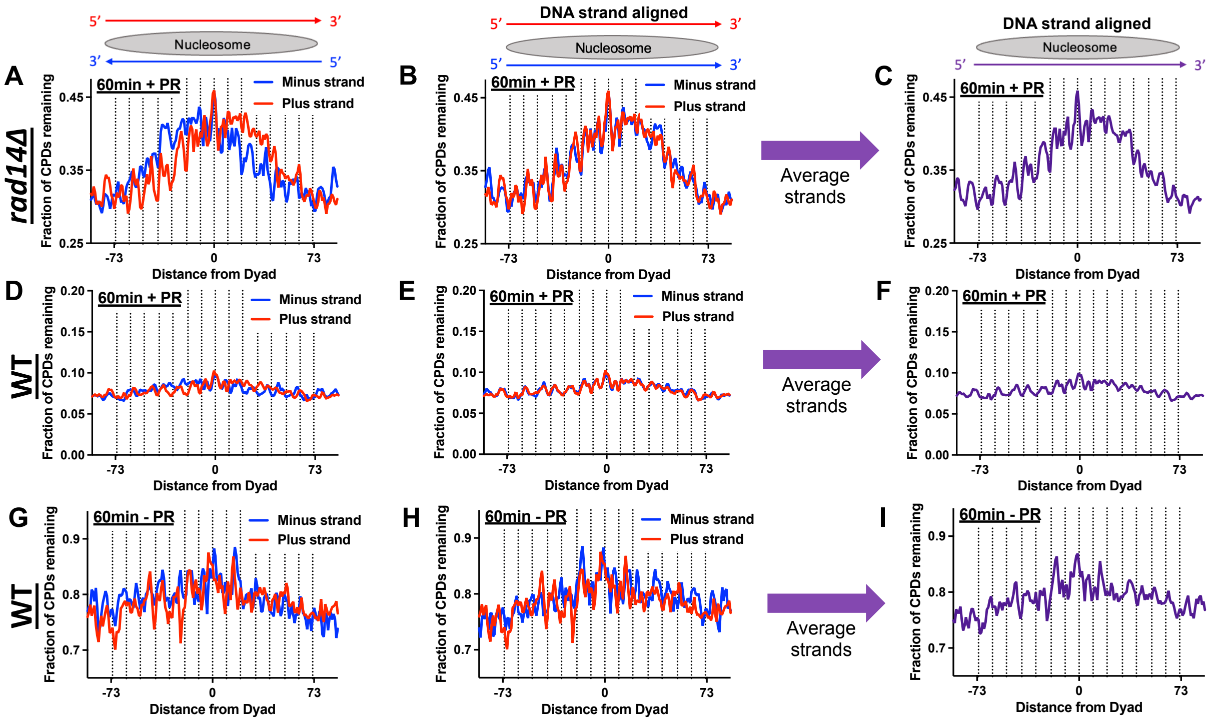

Figure S5: (A) Normalized fraction of CPDs remaining on both DNA strands after 60 minutes of photoreactivation (PR) following UVC irradiation of *rad14*Δ cells across ~10,000 strongly positioned nucleosomes (score > 5). Nucleosome coordinates are from (3). The central dyad axis of each nucleosomes indicated at position 0. Vertical dotted lines indicate minor-out rotational settings where the minor groove of the nucleosome DNA faces outward away from the histone octamer. (B) Same as panel A, except with the minus strand oriented in the 5’ to 3’ direction. (C) Same as panel B, except the aggregate of both DNA strands aligned in 5’ to 3’-orientation is plotted. (D) Fraction of CPDs remaining on both DNA strands after 60 minutes of both CPD photolyase repair and NER across ~10,000 strongly positioned nucleosomes (score > 5) within WT cells. (E) Same data as (D), with the minus strand oriented in the 5’ to 3’ direction. (F) Same data as (E), with the average of both 5’ to 3’-oriented DNA strands plotted. (G) Fraction of CPDs remaining on both DNA strands after 60 minutes of NER

across ~10,000 strongly positioned nucleosomes (score > 5) within WT cells. (H) Same data as (G), with the minus strand oriented in the 5' to 3' direction. (I) Same data as (H), with the average of both 5' to 3'-oriented DNA strands plotted. For all panels (A-I), nucleosome coordinates were from (3). Dotted lines at positions -73 and +73 bp from the dyad center represent the edges of the nucleosome core particle. Data plotted from -90 to -73 and +90 to +73 is within linker DNA.

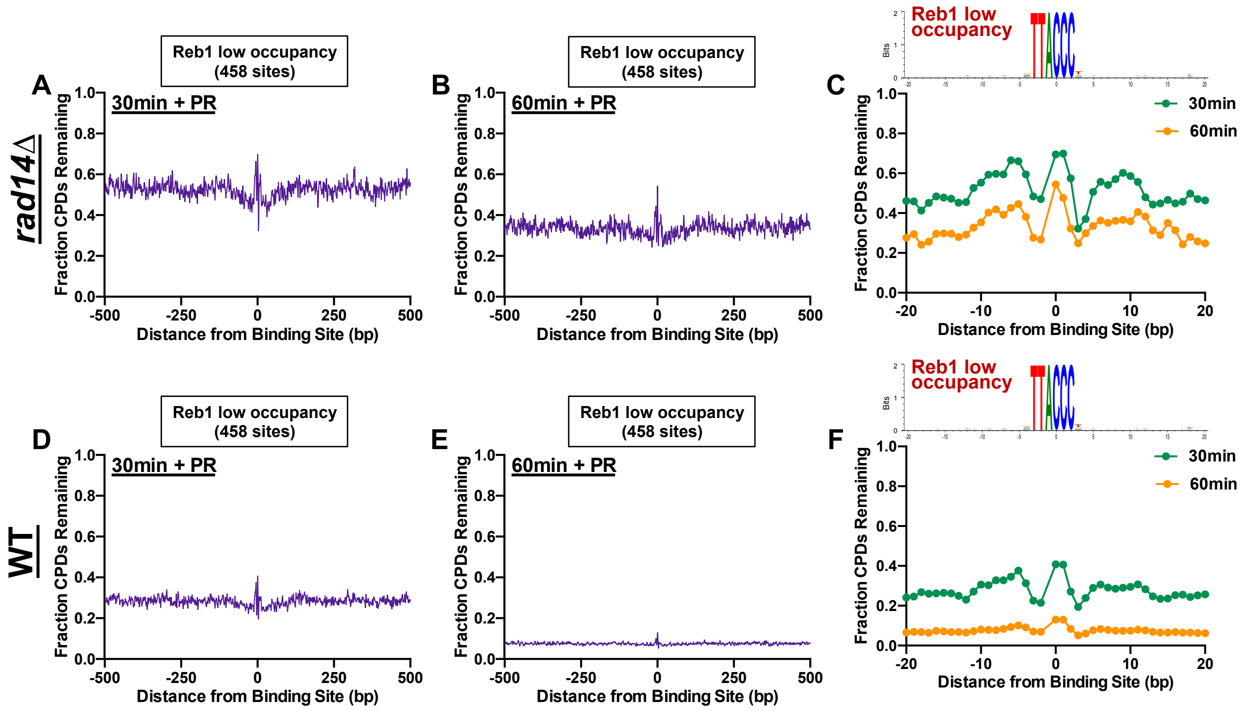

Figure S6: (A-B) Analysis of the fraction of CPDs remaining within 500 bp of the midpoint of 458 low occupancy (i.e., control) Reb1 binding sites following (A) 30min and (B) 60min of photoreactivation in *rad14*Δ cells. Reb1 binding site coordinates are from (4,5), except 1 redundant binding site was excluded. Fraction of CPDs remaining was normalized using the T4 endoV alkaline gel data. Positions associated with fewer than 10 CPDs in either time point were not plotted. (C) Closer examination of data shown in panels A,B. Distance spans -20 and +20 bp from center (position 0) of the low occupancy Reb1 binding motif. DNA sequence logo showing conserved nucleotides in the low occupancy binding motif is depicted at the top of the panel. Logo generated using weblogo3 software (6). (D-E) Same as panels A,B, except showing data for photoreactivation in NER-proficient WT cells. (F) Same as panel C, except for photoreaction of WT cells.

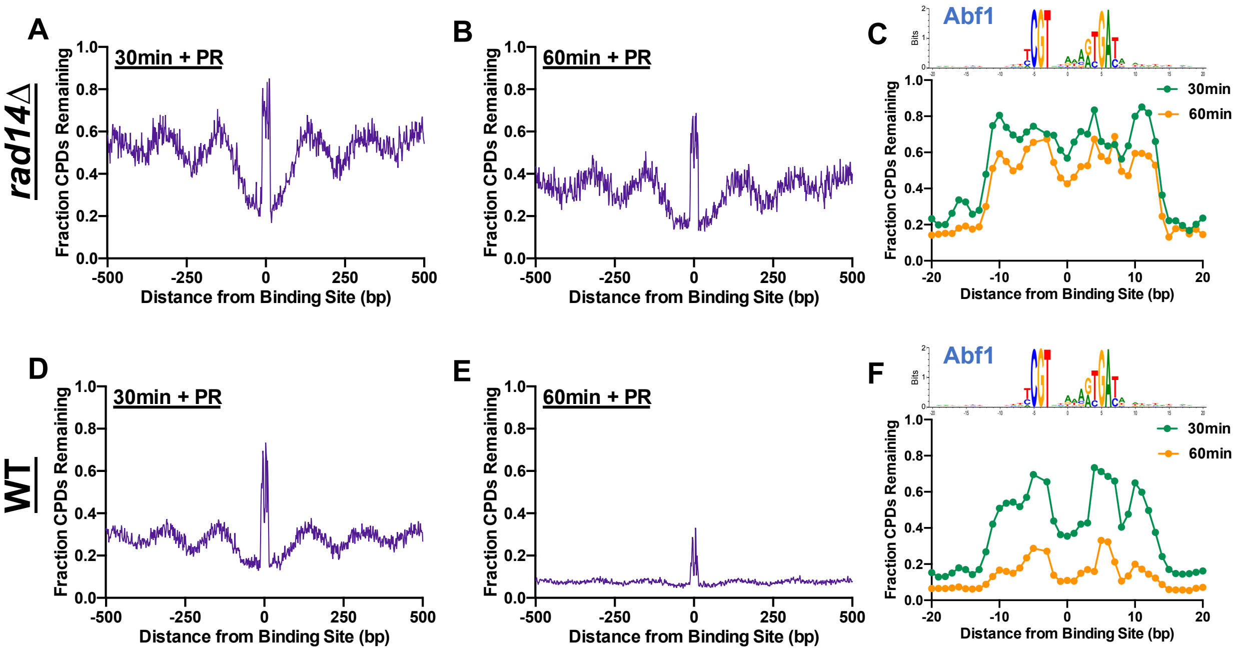

Figure S7: (A-B) Analysis of the normalized fraction of CPDs remaining within 500 bp of 280 Abf1 binding sites identified by ChIP-exo (7) following (A) 30min or (B) 60min of photoreactivation (PR) in UV-irradiated *rad14*Δ cells relative to the 0min control. Fraction of CPDs remaining was normalized using the T4 endoV alkaline gel data. Positions associated with fewer than 10 CPDs in either time point were not plotted. (C) Closer examination of data shown in panels A,B. Distance spans -20 and +20 bp from center (position 0) of the Abf1 binding motif. DNA sequence logo showing conserved nucleotides in Abf1 binding motif is depicted at the top of the panel. Logo generated using weblogo software (6). (D-E) Same as panels A,B, except showing data for photoreactivation in NER-proficient WT cells. (F) Same as panel C, except for photoreaction of WT cells.

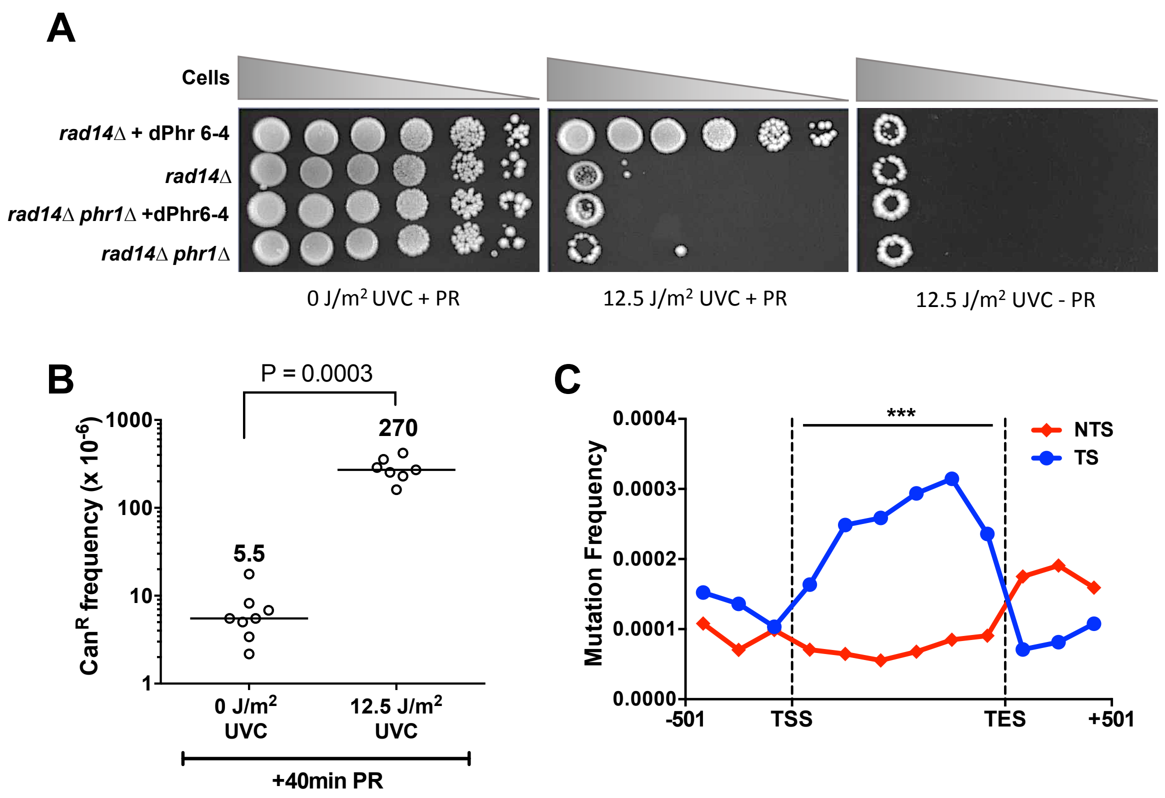

Figure S8: (A) UV sensitivity of NER-deficient *rad14*Δ cells with (top row) both endogenous CPD photolyase (*PHR1*) and *Drosophila* 6-4PP photolyase (dPhr6-4); second row, CPD photolyase alone; third row, dPhr6-4 alone; bottom row, no photolyases. (B) Frequency of canavanine resistant mutants in NER-deficient *rad14*Δ cells with endogenous CPD photolyase (*PHR1*) and *Drosophila* 6-4PP photolyase (dPhr6-4) exposed to the indicated doses of UVC light and photoreactivated (PR) for 40 minutes (40min). (C) Frequency of UV-induced mutations on each DNA strand (related to Fig. 6D) relative to the frequency of pyrimidine bases on each DNA strand. ***$P < 0.001$, based on a paired t-test for the difference in the frequency of UV-induced mutations between the TS relative to the NTS for the six coding region bins.

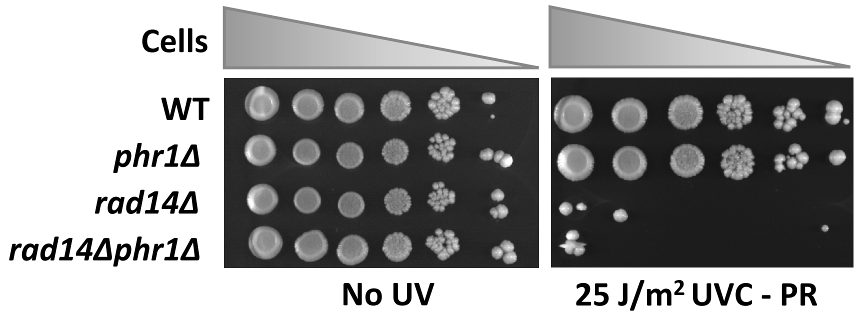

Figure S9: Sensitivity of WT yeast and yeast mutants deficient in photolyase (*phr1*Δ), NER (*rad14*Δ), or NER and CPD photolyase (*rad14*Δ*phr1*Δ) to a higher dose of UVC. Following UV exposure, all plates were incubated in darkness at 30°C and photographed.

Supplemental References

1. Holstege, F.C., Jennings, E.G., Wyrick, J.J., Lee, T.I., Hengartner, C.J., Green, M.R., Golub, T.R., Lander, E.S. and Young, R.A. (1998) Dissecting the regulatory circuitry of a eukaryotic genome. *Cell*, **95**, 717-728.
2. Weiner, A., Hsieh, T.H., Appleboim, A., Chen, H.V., Rahat, A., Amit, I., Rando, O.J. and Friedman, N. (2015) High-resolution chromatin dynamics during a yeast stress response. *Molecular cell*, **58**, 371-386.
3. Brogaard, K., Xi, L., Wang, J.P. and Widom, J. (2012) A map of nucleosome positions in yeast at base-pair resolution. *Nature*, **486**, 496-501.
4. Mao, P., Smerdon, M.J., Roberts, S.A. and Wyrick, J.J. (2016) Chromosomal landscape of UV damage formation and repair at single-nucleotide resolution. *Proceedings of the National Academy of Sciences of the United States of America*, **113**, 9057-9062.
5. Kasinathan, S., Orsi, G.A., Zentner, G.E., Ahmad, K. and Henikoff, S. (2014) High-resolution mapping of transcription factor binding sites on native chromatin. *Nature methods*, **11**, 203-209.
6. Crooks, G.E., Hon, G., Chandonia, J.M. and Brenner, S.E. (2004) WebLogo: a sequence logo generator. *Genome research*, **14**, 1188-1190.
7. Rossi, M.J., Kuntala, P.K., Lai, W.K.M., Yamada, N., Badjatia, N., Mittal, C., Kuzu, G., Bocklund, K., Farrell, N.P., Blanda, T.R. *et al.* (2021) A high-resolution protein architecture of the budding yeast genome. *Nature*, **592**, 309-314.
